# Closing in on human methylation—the versatile family of seven-β-strand (METTL) methyltransferases

**DOI:** 10.1093/nar/gkae816

**Published:** 2024-10-01

**Authors:** Pål Ø Falnes

**Affiliations:** Department of Biosciences, University of Oslo, PO Box 1066 Blindern, 0316Oslo, Norway; CRESCO - Centre for Embryology and Healthy Development, University of Oslo and Oslo University Hospital, Oslo, Norway

## Abstract

Methylation is a common biochemical reaction, and a number of methyltransferase (MTase) enzymes mediate the various methylation events occurring in living cells. Almost all MTases use the methyl donor S-adenosylmethionine (AdoMet), and, in humans, the largest group of AdoMet-dependent MTases are the so-called seven-β-strand (7BS) MTases. Collectively, the 7BS MTases target a wide range of biomolecules, i.e. nucleic acids and proteins, as well as several small metabolites and signaling molecules. They play essential roles in key processes such as gene regulation, protein synthesis and metabolism, as well as neurotransmitter synthesis and clearance. A decade ago, roughly half of the human 7BS MTases had been characterized experimentally, whereas the remaining ones merely represented hypothetical enzymes predicted from bioinformatics analysis, many of which were denoted METTLs (METhylTransferase-Like). Since then, considerable progress has been made, and the function of > 80% of the human 7BS MTases has been uncovered. In this review, I provide an overview of the (estimated) 120 human 7BS MTases, grouping them according to substrate specificities and sequence similarity. I also elaborate on the challenges faced when studying these enzymes and describe recent major advances in the field.

## Graphical Abstract

**Graphical Abstract:**
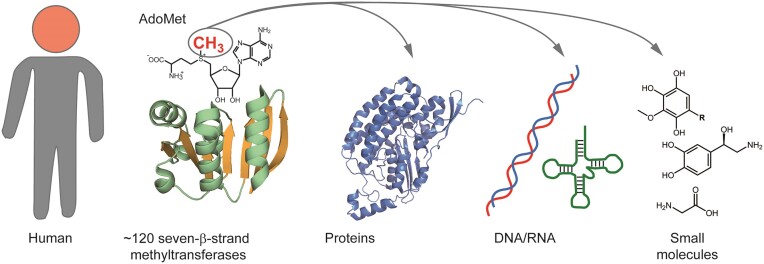

## Introduction

**Figure 1.**
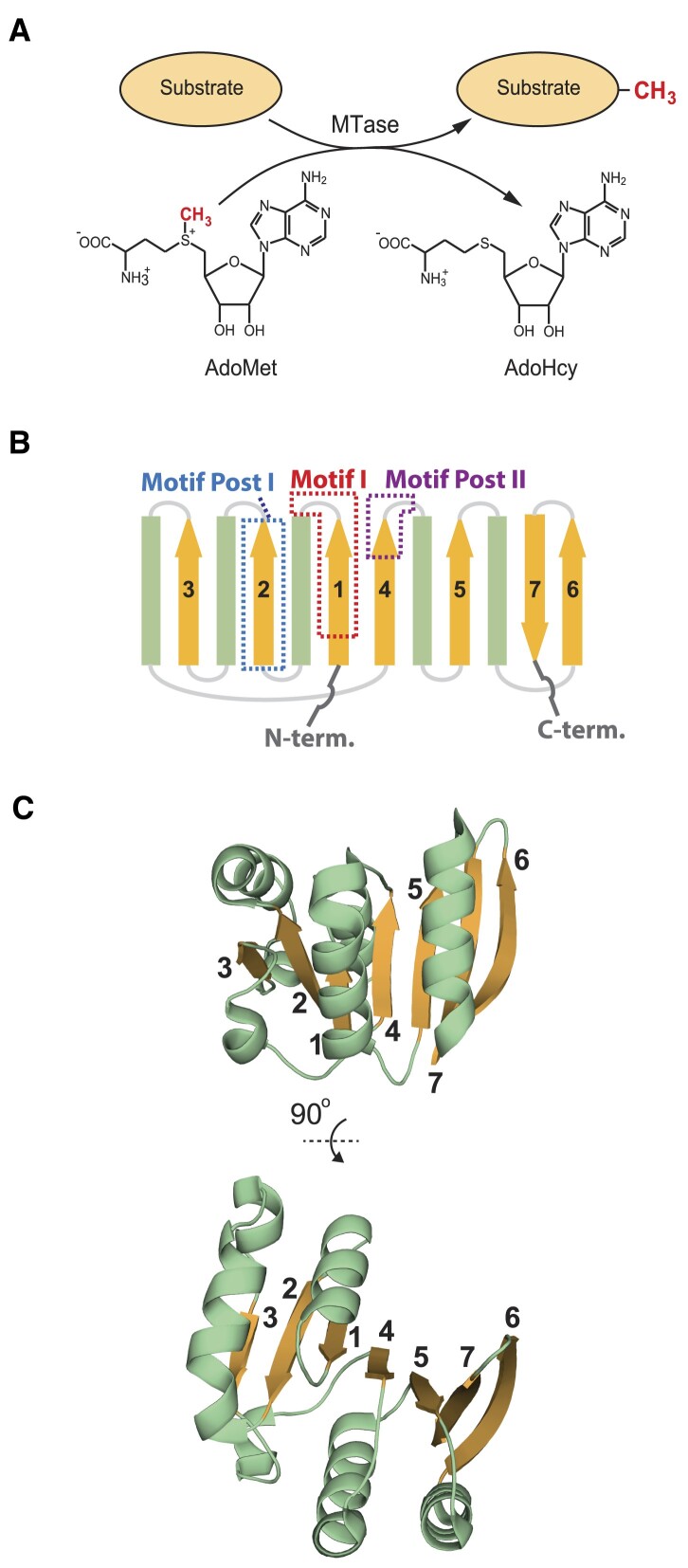
The 7BS MTase reaction and fold. (**A**) The *S*-adenosylmethionine (AdoMet)-dependent MTase reaction. The MTase catalyses the transfer of a methyl group from AdoMet to the substrate, yielding *S*-adenosylhomocysteine (AdoHcy) and methylated substrate. (**B**) Topology of the canonical 7BS MTase fold. Key motifs involved in AdoMet (Motif I and Motif Post I) and substrate (Motif Post II) binding are indicated. The β-strands are shown in orange and numbered; α-helices are shown in green. (**C**) Three-dimensional structure of a typical 7BS MTase fold. A cartoon representation of the 7BS region (amino acids 68 -211) of the lysine-specific MTase METTL21A is shown. Colouring and numbering is as in (B). The structure (PDB: 4LEC) was visualized using PyMol (https://pymol.org/).

In cells, a number of methyltransferases (MTases) catalyse the attachment of methyl groups to various substrate molecules. In these methylation reactions, a methyl group is transferred from a methyl donor, usually *S*-adenosylmethionine (AdoMet), to the relevant substrate, leaving *S*-adenosylhomocysteine (AdoHcy) as a byproduct (Figure 1A). In humans, there are ∼200 different MTases, which are subdivided into distinct classes based on their structure (1). Of these, the seven-β-strand (7BS; also called ‘Class I’ or ‘Rossmann-like’) MTase class is the largest, comprising ∼120 AdoMet-dependent MTases (1,2). The second largest MTase class in humans is the SET-domain proteins, comprising ∼55 MTases that are not covered by this review (3). The SET-domain MTases mostly methylate lysines in histones and other proteins, and play key roles in regulating gene expression and chromatin structure (3).

Collectively, the 7BS MTases target a multitude of substrates, and, based on their substrate specificity, they can be subdivided into the three following groups: protein MTases, DNA/RNA MTases, and small molecule (SM) MTases, which target various metabolites, neurotransmitters and toxic compounds. Overall, 7BS MTase-mediated methylation plays important roles in diverse cellular processes, including metabolism, protein synthesis, neural signaling, and gene regulation at both the epigenetic/transcriptional and epitranscriptomic/post-transcriptional levels. In this review, I will give an inventory and overview of the various types of 7BS MTases, particularly emphasizing recent discoveries. Also, I will discuss some of the more general features of these enzymes and address some of the challenges faced when studying them.

## The 7BS MTase fold

The 7BS MTases share a three-dimensional fold where the core component is a twisted seven-stranded β-sheet of a distinct topology, and the individual β-strands are organized in a specific order and direction, with all except the last strand being parallel (Figure 1B and C). Furthermore, the 7BS MTase fold consists of alternating (throughout the linear protein sequence) β-strands and α-helices, with α-helixes at both sides of the β-sheet, thus forming the three dimensional structure of an αβα sandwich. Several characteristic, yet degenerate sequence motifs are found at distinct locations within the 7BS fold (4–6) (Figure 1B). Of these, ‘Motif I’ and ‘Motif Post I’ contain invariant residues critical for AdoMet binding. ‘Motif Post II’, which contains amino acid residues involved in substrate binding, shows little conservation across the entire 7BS MTase superfamily, but is typically conserved between related MTases that target similar substrates, as well as between orthologous MTases from different organisms (7).

Although most 7BS MTases adhere rather strictly to the canonical 7BS fold, there are also some exceptions, i.e. MTases that have a different Motif I sequence or a slightly deviating topology (1,2). For example, the protein arginine methyltransferases (PRMTs) lack β-strands 6 and 7 (8), and some *N*^6^-adenine MTases have a different order and/or number of β-strands (9–11). Also, certain non-MTase 7BS proteins that use AdoMet-like cofactors are largely indistinguishable from the 7BS MTases based on sequence and structure. For example, the related spermidine and spermine synthetases catalyse the transfer of an aminopropyl moiety from decarboxylated AdoMet to relevant substrates during polyamine synthesis (12). Moreover, a few 7BS proteins are devoid of independent MTase activity, but associate with paralogous 7BS MTases, enhancing their activity. This is the case for DNMT3L, which is an accessory factor for the DNA MTases DNMT3A and DNMT3B, and for METTL14, which forms a complex with the RNA MTase METTL3 (13,14). Note that, despite lacking intrinsic MTase activity, DNMT3L and METTL14 have been included in the current compilation of 7BS MTases.

## A brief history of mammalian 7BS MTase research

**Figure 2.**
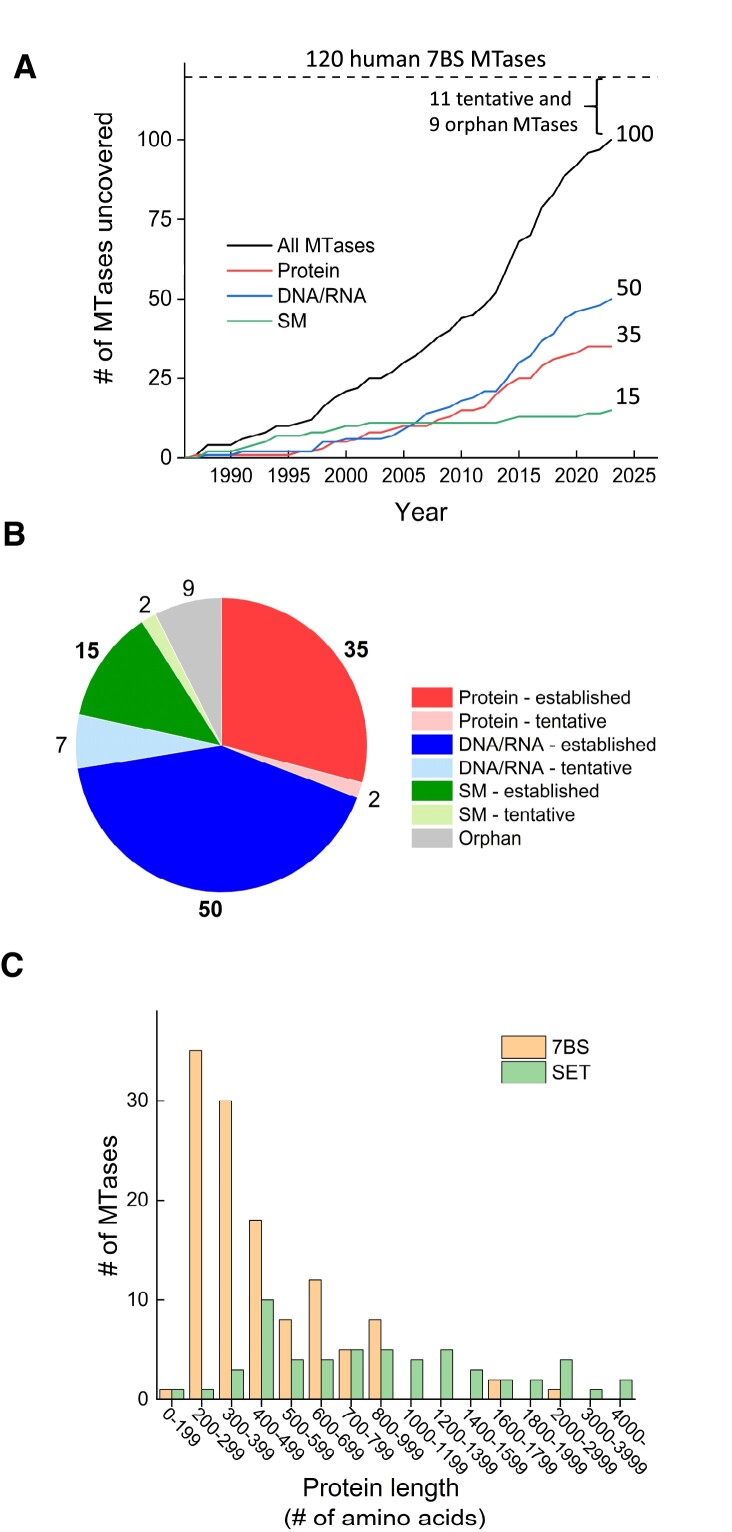
Discovery, groupings and current status of human seven-β-strand methyltransferases (7BS MTases). The MTases have been placed in three groups according to their substrates, i.e. protein (red), DNA/RNA (blue) or small molecule (SM; green). Furthermore, characterized MTases are denoted as ‘established’, whereas uncharacterized MTases are denoted as either ‘tentative’ or ‘orphans’. For the tentative MTases, the substrate type (protein, DNA/RNA or SM) may be inferred from similarity to known MTases, while for the orphan MTases, the substrate type remains elusive. (**A**) Timeline for the discoveries of human 7BS MTases. The year of discovery indicates the time at which both the MTase activity and encoding gene had been uncovered. The current number of characterized MTases within each group is indicated by the number at the end of each curve. (**B**) Pie chart indicating the characterization status of the MTases as well as their distribution between the groups. Color code and groupings are as in (A), but with the tentative MTases indicated by pale colour. The orphan MTases are depicted in grey. The information used to generate the plots in (A) and (B) can be found in Supplementary Tables S1–S3. (**C**) Comparison of length distributions between 7BS MTases and SET-domain MTases. Information on the lengths of the 120 7BS MTases and 56 SET domain MTases in humans was obtained from UniProt.

The first discovered mammalian 7BS MTases methylate various small molecules (SM), and the majority of such SM MTases were identified during the rather early years of MTase research, i.e. during the 1950s and 1960s. Typically, the SM MTases were initially described as biochemical activities in tissue extracts, later followed by the purification and protein sequence determination of the corresponding enzymes. However, it would take many years before the encoding genes were cloned and their sequence determined. In contrast, the majority of protein and nucleic acid MTases were discovered much later, mainly from the year 2000 and onwards, aided by data from the sequencing and annotation of the human genome. This is illustrated by Figure 2A, which shows a timeline for the complete characterization (gene identified and MTase activity determined) of the human 7BS MTases, showing also separate timelines for the three MTase groups (protein, DNA/RNA and SM).

The SM MTase nicotinamide *N*-methyltransferase (NNMT) represented the first described MTase activity, which was observed in partially purified rat liver extracts by Cantoni as early as 1951 (15). One year later, AdoMet was found, also by Cantoni, to function as the methyl donor (16). Pioneering work was done also by Axelrod, who in 1957 described catecholamine methyltransferase (COMT) activity, and used radiolabeled AdoMet, still a widely used reagent for MTase research to this day, to later discover acetylserotonin O-methyltransferase (ASMT) (17–19).

As with the SM MTases, mammalian protein and nucleic acid MTase activity was first described in cell or tissue extracts, but it should be noted that some of these activities were later attributed to SET domain MTases, rather than to 7BS MTases. RNA MTase activity was described already in 1963 by Srinivasan and Borek, who later went on to also report DNA MTase activity (20,21). Finally, protein MTase activity was demonstrated by Paik and Kim in 1968 (22). However, the vast majority of the actual MTases were discovered much later by using reverse genetics approaches. They were first annotated as theoretical proteins encoded by the human genome, and subsequently, based on sequence, annotated as putative MTases, some of which were denoted as METTLs (methyltransferase like proteins).

Later, these putative MTases were functionally characterized, typically through investigations on the biochemical activity of the recombinant protein and/or on the effects of MTase ablation on the methylation status of relevant target molecules. These studies benefited greatly from important technical advances, such as improved mass spectrometry (MS) and liquid chromatography (LC) analyses, as well as novel gene targeting technologies. Also, our understanding of many of these human MTases was aided by previous characterizations of their orthologues/homologues in the budding yeast *Saccharomyces cerevisiae*. For example, the first human 7BS lysine (K)-specific MTase (KMT) DOT1L was identified based on studies of its yeast orthologue Dot1, and several human tRNA MTases are denoted as TRMTs, reflecting their similarity/orthology to corresponding, previously identified yeast ‘Trm’ (tRNA MTase) proteins.

The first three-dimensional structure of an MTase was published in 1993, when Cheng et al. showed that the bacterial restriction MTase HhaI adopts what is now appreciated as the characteristic 7BS MTase architecture (23). This was followed by the publication of a similar structure of mammalian COMT shortly thereafter (24), and in the following years, the 7BS fold was found in many additional MTase structures. It was also noted that the 7BS MTases shared distinct sequence motifs found at distinct positions in the structure (4,6).

## An inventory and overview of human 7BS MTases

A hallmark bioinformatics study by Petrossian and Clarke from 2011 used sequence and secondary structure features to predict the full complement of human MTases, i.e. the human ‘methyltransferasome’, and estimated the existence of 131 human 7BS MTases, represented by corresponding UniProt entries (1). Here, I have updated this number to 120, encompassing only reviewed (Swiss-Prot) entries. Obsolete entries and likely pseudogenes were removed, as well as the 7BS proteins later shown to have non-MTase functions. Moreover, the DNA MTases DNMT3A, -3B and 3L (which were omitted originally), as well as the recently discovered RNA MTase PCIF1/CAPAM and the uncharacterized MTase METTL24, were added.

While only about 50% of the human 7BS MTases had been characterized by 2010, the corresponding number is now well above 80% (Figure 2A). In particular, several novel protein and RNA MTases have been discovered in recent years, and the 100 characterized MTases now comprise 50 DNA/RNA MTases, 35 protein MTases and 15 SM MTases (Figure 2A and B). The 20 yet uncharacterized MTases have been categorized either as ‘tentative’ (11 MTases), where the type of substrate (protein, DNA/RNA or SM) may be tentatively inferred from sequence similarity to established MTases, or as ‘orphans’ (9 MTases), whose substrate type remains elusive. Notably, a large portion of the human 7BS MTases are relatively small proteins with few or no additional annotated domains in addition to the MTase domain. This is illustrated by a comparison with the SET domain MTases, which typically have additional domains involved in recruitment to or modification of chromatin (Figure 2C). Generally, the 7BS MTases are much smaller that the SET domain proteins, with ∼80% of the MTases ranging from 200 to 500 amino acids in length (Figure 2C).

**Figure 3.**
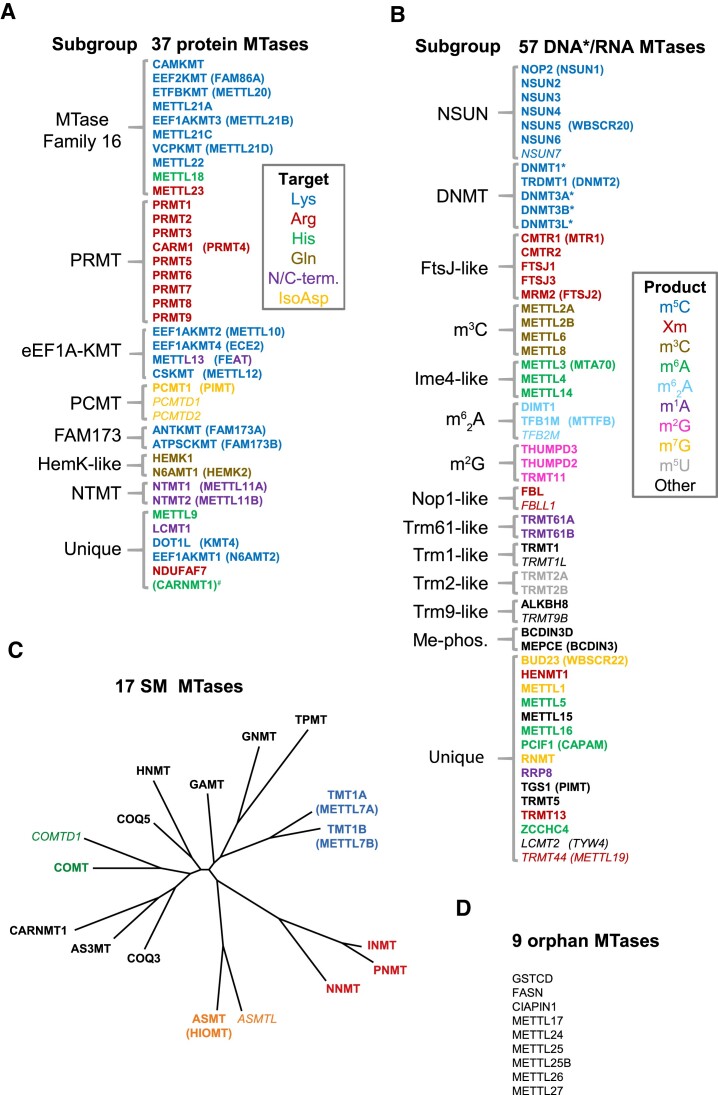
An inventory and overview of the human 7BS MTases. The MTases are indicated by their HGNC gene symbols, in some cases with alias symbols in parenthesis. In **(**A–C), established MTases are in bold print, whereas tentative MTases are in italics. In (A) and (B), the MTases have been placed into subgroups based on sequence similarity, and those that do not show appreciable similarity to others are designated as ‘unique’. (**A**) Subgroupings and substrates of protein MTases. Colors indicate the substrate types that are targeted by the MTases. PRMT, protein arginine MTase group; eEF1A-KMT, eukaryotic elongation factor 1α lysine MTase group; PCMT, protein isoaspartate (IsoAsp) MTase group; FAM173, Family With Sequence Similarity 173 group; HemK-like, similar to HemK from bacteria; NTMT, N-terminal MTase subgroup. ^#^Note that CARNMT1, which first was shown to be a SM MTase, but was later established also as a protein MTase, is not counted as one of the 37 protein MTases, and thus placed in parenthesis; this is to avoid discrepancies with the numbers in Figure 1. (**B**) Subgroupings and products of DNA/RNA MTases. The DNA MTases are indicated by an asterisk (*). Colors indicate the products that are generated by the MTases. In most cases, the subgroups have been given names from bacterial (FtsJ) or yeast (Ime4, Nop1, Trm1, Trm2, Trm9, Trm61) homologues/orthologues, or the subgroup name indicates the methylated product formed. NSUN, NOL1/NOP2/SUN domain subgroup; DNMT, DNA MTase subgroup; Me-phos., 5′ methylphosphate-generating subgroup. (**C**) Unrooted phylogenetic tree of human small molecule (SM) MTases. MTases that display sequence similarity (beyond the generic 7BS motifs) are indicated by the same colour (blue, red, green or orange). (**D**) Orphan MTases. This group represents uncharacterized human 7BS MTases without tentative substrates.

Based on the three substrate-based groups (protein, DNA/RNA and SM), I will in the following sections give an account of the human 7BS MTases, emphasizing recent discoveries. An overview of all the 120 MTases, grouped according to substrate type, is given in Figure 3. The MTases are here denoted by their approved HGNC (HUGO Gene Nomenclature Committee; https://www.genenames.org/) gene symbols, and more information can therefore readily be retrieved from the HGNC website and links therein (e.g. to UniProt). However, some of the lysine specific MTases (KMTs) are in the text referred to by a more readable, near-identical variant of the symbol (including lowercase and Greek letters, as well as dashes; e.g. eEF1A-KMT1 and ETFβ-KMT instead of EEF1AKMT1 and ETFBKMT, respectively). Also, more detailed information on the MTases and their substrates can be found in Supplementary Tables S1 (protein MTases), S2 (DNA/RNA MTases) and S3 (SM MTases). Importantly, the main groupings used here (protein, DNA/RNA, SM, and orphan 7BS MTases) are also used by the HGNC in their revised organization of this gene family (‘Seven-beta-strand methyltransferase motif containing’)(https://www.genenames.org/data/genegroup/#!/group/1400)(25).

**Figure 4.**
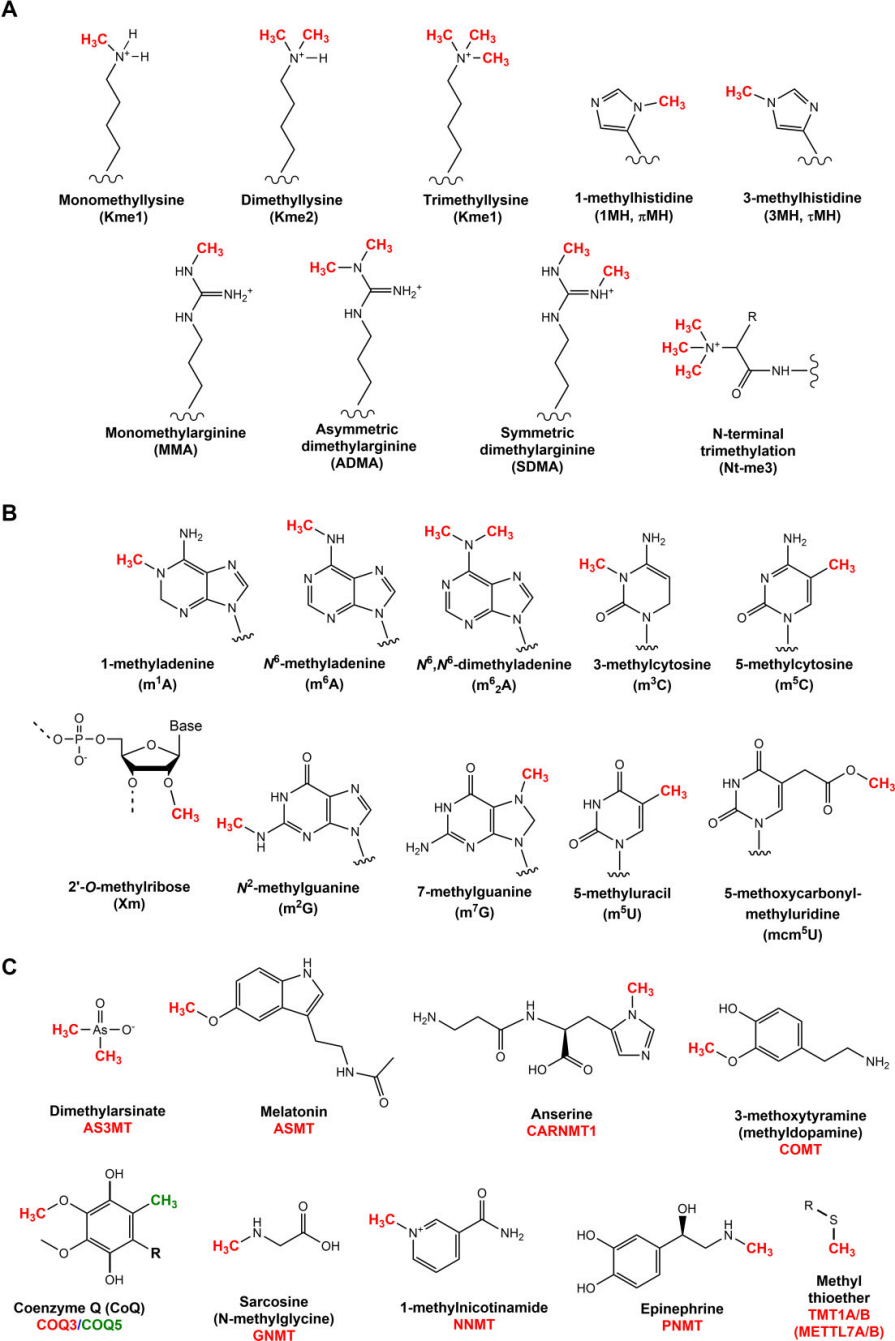
Products of 7BS MTase-catalysed reactions. Key products formed by 7BS MTase-mediated transfer of methyl groups (red or green) onto proteins (**A**), RNA/DNA (**B**) and small molecules (**C**). In (B), the abbreviations refer to the shown nucleobases (and not to RNA nucleosides, which is more common); this is because some of the methylated bases are discussed in the context of both DNA and RNA. In (C), the gene symbols of the enzymes responsible for the methylations are also indicated, i.e. AS3MT (arsenite MTase), ASMT (acetylserotonin *O*-MTase), CARNMT1 (carnosine *N*-MTase), COMT (catechol *O*-methyltransferase), COQ3 (ubiquinone biosynthesis *O*-MTase), COQ5 (ubiquinone biosynthesis MTase), GNMT (glycine *N*-MTase), NNMT (nicotinamide *N*-MTase), PNMT (phenylethanolamine *N*-MTase), TMT (thiol *S*-MTase).

As the sequence similarity between different human 7BS MTases is typically very low, it is difficult to construct meaningful phylogenetic trees based on their alignments. However, most of the MTases show substantial sequence similarity, typically detectable by standard protein BLAST searches, to one or several of the others, especially in the substrate-recognizing Motif Post II (Figure 1B). Thus, they form subgroups of paralogous MTases, each with 2–10 members, whereas the remaining ∼1/3 of the MTases are ‘unique’, not showing substantial similarity to other MTases. The protein and DNA/RNA MTases have here been organized into such subgroups (Figure 3A and B), often named either after putative orthologues from yeast (e.g. Ime4, Nop1 and Trm's) or bacteria (e.g. HemK, FstJ), and the members of a subgroup typically target similar substrates. The smaller SM MTase group is illustrated by a phylogenetic tree (Figure 3C), whereas the orphan MTases are shown as a list (Figure 3D). Finally, important methylated products generated by the 7BS MTases are shown in Figure 4.

## Protein MTases

Most of the human 7BS protein MTases are found in subgroups whose members mainly target the same amino acid (Figure 3). For example, arginine methylation is primarily mediated by members of the protein arginine MTase (PRMT) family of related MTases, whereas most of the members of the so-called MTase Family 16 (MTF16) are lysine-specific MTases (KMTs). However, there are also several unique protein MTases. The protein methylations mediated by the 7BS MTases appear rather static and non-reversible, as no corresponding demethylase has been identified. This is in stark contrast to the SET domain MTases, which represent the second largest human MTase family and commonly target lysines in histones (3); for these, a number of antagonizing demethylases yield dynamicity and regulatory potential (26).

### Lysine-specific MTases (KMTs)

The ϵ-amino group of lysine can receive up to three methyl groups, yielding the three distinct methylation states; mono-, di-, and tri-methyllysine (Figure 4A). In 2002, DOT1L was found to methylate Lys-79 of the globular domain of histone H3, thus representing the first human 7BS KMT (27–29). Several years later, in 2010, the long-sought KMT targeting Lys-115 in calmodulin (CaM) was identified and named CaM-KMT (gene name: *CAMKMT*) (30), and then found to belong to a distinct 7BS MTase family/subgroup, Methyltransferase Family 16 (MTF16; also denoted ‘Group J’) (1,31,32).

Presently, 16 distinct human 7BS KMTs have been reported, eight of which belong to the MTF16 subgroup (33). These KMTs are highly specific, with all evidence to date indicating that they methylate a single substrate protein (and, in some cases, its paralogues), which in most cases belongs to one of the following groups: molecular chaperones, mitochondrial proteins, and components of the translational machinery (33). The key chaperones Hsp70 and VCP/p97 are methylated by the dedicated KMTs METTL21A (31,34) and VCP-KMT (31,32), respectively. Moreover, distinct KMTs target important mitochondrial proteins, i. e. citrate synthase (CS-KMT) (35,36), the β-subunit of the electron transfer flavoprotein (ETFβ-KMT) (37,38), the adenosine nucleotide transporter (ANT-KMT) (39) and the c-subunit of the ATP synthase (ATPSc-KMT) (40). Several key components of the translational machinery are subject to 7BS-KMT-mediated lysine methylation, i.e. the universally conserved eukaryotic elongation factors eEF1A (there are two paralogues in humans, eEF1A1 and eEF1A2) and eEF2, as well as the alanine aminoacyl transferase (AARS1). Notably, five distinct lysines in eEF1A are methylated by dedicated individual KMTs (eEF1A-KMT1-4 and METTL13) (41–47), whereas single lysines in eEF2 and AARS1 are subject to methylation by eEF2-KMT (48) and METTL21C (49), respectively.

### Arginine MTases (PRMTs)

In eukaryotes, arginine is frequently methylated, and the two terminal N-atoms of its guanidine group can jointly accept up to two methyl groups. This yields three different forms of methylated arginine, i.e. monomethylarginine (MMA), as well as two types of dimethylarginine, i.e. asymmetric dimethylarginine (ADMA) with both methyl groups placed at the same N-atom, and symmetric dimethylarginine (SDMA) where the two N-atoms receive one methyl group each (Figure 4A)(50). The vast majority of arginine methylation in humans is mediated by nine related protein arginine MTases (PRMTs) that form a distinct MTase subfamily. Based on their enzymatic activities, PRMTs are placed in three different groups; type I (PRMT1, PRMT2, PRMT3, PRMT4, PRMT6, PRMT8), type II (PRMT5 and PRMT9) and type III (PRMT7). The type I and II PRMTs catalyse the formation (via MMA) of ADMA and SDMA, respectively, whereas the type III enzyme PRMT7 only generates MMA (50,51).

Several of the PRMTs introduce methylations in the flexible tails of histone proteins, which contribute to the regulation of chromatin function, but PRMTs also target many non-histone substrates, such as components of the machineries for transcription, mRNA splicing and DNA repair (51). While some PRMTs are broad-specificity enzymes, targeting short sequences, such as arginine-and glycine-rich motifs (denoted RGG/RG motifs), others are more specific and methylate only one or a few substrates (50). Most of the PRMTs were uncovered and characterized several years ago, with PRMT9, which was unravelled in 2015, as the most recent addition (52). More recently, the 7BS MTases METTL23 and NDUFAF7, which are unrelated to the nine canonical PRMTs, were reported to methylate distinct arginines in histone H3 and the mitochondrial protein NDUFS2, respectively (53,54). However, these substrate assignments could benefit from more biochemical evidence.

### Histidine MTases

Histidines can be methylated at the N1 (π) or N3 (τ) positions, yielding 1-methylhistidine (1MH, πMH) or 3-methylhistidine (3MH, τMH), respectively (Figure 4A). Even though protein histidine methylation was discovered more than 50 years ago, the first human protein histidine MTase was discovered only recently, when it was shown that the SET-domain MTase SETD3 introduces 3MH in actin (55,56). Since then, three additional histidine MTases, which all belong to the 7BS family, have been identified. METTL9 introduces 1MH at His-X-His motifs in various proteins (57), whereas METTL18 specifically introduces a single 3MH modification in the ribosomal protein RPL3 (58,59). Finally CARNMT1, which previously was found to catalyse the formation of 1MH in the context of the dipeptide carnosine, was recently reported to also be a protein MTase, introducing 1MH in a number of proteins, usually within so-called C3H (3xCys-His) zinc fingers (60).

### Other protein MTases

Together, Arg, Lys and His methylation constitute the bulk of human protein methylation, but other types of protein methylation also exist. A universally conserved glutamine methylation in mitochondrial and cytosolic translation release factors is introduced by HEMK1 and N6AMT2/HEMK2, respectively; these are homologues of the bacterial MTase HemK/PrmC (61–63). Also, several MTases (NTMT1, NTMT2, LCMT1, METTL13) target the N- or C-termini of proteins (44,64–66). The most recently discovered one, METTL13, is unique in that it carries two MTases domains, where one targets the N-terminus and the other an internal lysine residue on the translation elongation factor eEF1A (44,45). Finally, PCMT1 is a protein repair enzyme which methylates spontaneously arising l-isoaspartate residues, thus aiding their reversal to Asp (67).

## DNA/RNA MTases

RNA is subject to numerous post-transcriptional modifications. The majority of these are methylations, and, accordingly, almost half of the human 7BS MTases target RNA. The corresponding methylations are found in virtually all RNA species, including ribosomal RNA (rRNA), mRNA and tRNA, as well as microRNAs and other non-coding small RNAs. 5-methylcytosine (m^5^C) is an essential modification in mammalian DNA, and the possible existence of other, biologically important methylations in human DNA remains a matter of debate.

### *N^6^*-adenine MTases

*N^6^*-Methyladenine (m^6^A) is a highly abundant modification in mRNA, introduced at specific sequence motifs by an MTase complex containing METTL3 and METTL14 (14). Also, m^6^A in mRNA is highly dynamic and regulates many aspects of mRNA function and biogenesis, including splicing, nuclear export, translation and stability (68). Central are reader proteins and demethylases that recognize and remove m^6^A, respectively, similarly to how epigenetic modifications on histone proteins are regulated. Thus, m^6^A and other RNA modifications are frequently referred to as ‘epitranscriptomic’, although for many of these modifications, a regulatory role remains to be established. Two additional mRNA-targeting *N*^6^-adenosine MTases have been reported. METTL16, which methylates a specific A residue in U6 snRNA, was also shown to target similar structures in mRNA (69,70). The MTase PCAF1/CAPAM targets the first transcribed nucleotide in the mRNA when it is an adenosine, converting it to m^6^A (11).

Several additional m^6^A MTases that target non-mRNA species were recently identified. It was known for many years that the two large ribosomal RNAs (rRNAs), 18S and 28S rRNA, contain one m^6^A modification each, and the corresponding MTases were recently identified as METTL5 and ZCCHC4, respectively (71–73). Similarly, METTL4 was recently identified as the long-sought MTase responsible for a *N*^6^-methylation of a single, also 2′-*O*-ribose-methylated (Am) residue in the U2 small nuclear RNA (snRNA) (74,75).

During the last decade it has been demonstrated that m^6^A is an important epigenetic DNA modification in many eukaryotes, such as the green algae *Chlamydomonas* (76). Several studies have also reported the presence of m^6^A in mammalian DNA, as well as the responsible MTases, but many of the results have been questioned and suggested to be influenced by experimental artifacts (77,78). Thus, it may be too early to conclude that any of the human 7BS MTases (such as N6AMT2 and METTL4; see below) play any biologically significant role in the m^6^A modification of DNA. Importantly, methylations on nucleic acids are, besides being installed by specific MTases, also generated spuriously and non-enzymatically by various methylating agents (including AdoMet), thus representing damage that may be difficult to distinguish from useful methylations. For example, 1-methyladenosine (m^1^A), which actually can be converted to m^6^A by a so-called Dimroth rearrangement (79), is an important RNA modification, introduced by several dedicated human 7BS MTases (Figure 3). However, m^1^A is also a significant replication-blocking lesion in DNA, which is repaired by specific demethylases of the AlkB family (80,81). Actually, the human AlkB homologs FTO and ALKBH5 mediate the reversal of m^6^A modifications in mRNA (82,83), thus further illustrating the interconnection between harmful and useful methylations on nucleic acids.

### C^5^-cytosine MTases

In human DNA, m^5^C is mostly found at CG motifs, so-called CpGs, and it plays a central role in gene repression and the formation of inactive heterochromatin states. The MTases responsible for installing m^5^C in mammalian DNA all belong to the DNMT (DNA methyltransferase) family of related enzymes (84). DNMT3A and DNMT3B are responsible for de novo formation of m^5^C, whereas DNMT1 targets hemimethylated DNA, and thus ensures that methylation states are maintained when the DNA is replicated (84). DNMT3L is devoid of DNMT catalytic activity but is an accessory factor that forms a heterodimeric complex with the DNMT3 enzymes, enhancing their catalytic efficiency (13). Clearly, studies of mammalian DNA methylation and the responsible DNMTs has had a tremendous impact on our understanding of gene regulation and various epigenetic phenomena, such as imprinting, X-chromosome inactivation and transposon silencing (85).

m^5^C is also highly abundant in RNA, where it is introduced by several different MTases. The fifth member of the human DNMT family, DNMT2 (now denoted TRDMT1), is actually an RNA MTase, introducing m^5^C at position 38 in the anticodon loop of some tRNAs (86). In addition, the members of the NOL1/NOP2/SUN (NSUN) MTase family are all C^5^-cytosine RNA MTases (87). NSUN1-6 target specific positions in tRNA and rRNA, both in the nucleus and mitochondria, whereas the function of the seventh member NSUN7 remains elusive (87).

### 2′-*O*-Ribose MTases

Methylation of the 2′-*O*-position in the ribose ring is a highly abundant RNA modification introduced by several distinct MTases (Figure 4B). 2′-*O*-methyl ribose (Xm) is particularly abundant in rRNA, where the MTase fibrillarin (FBL) uses small nucleolar RNAs (snoRNAs) as guides to introduce Xm at several positions. Collectively, the Xm MTases target a wide range of RNA species, including tRNA (FTSJ1, TRMT13) (88,89), piRNA (HENMT1) (90), rRNA (FBL, MRM2) (91,92), as well as the cap of mRNA (CMTR1, CMTR2). Interestingly, it was shown that FTSJ3, which plays a still elusive role in rRNA maturation (93), is important during HIV infection (94,95). FTSJ3 is hijacked by interacting viral proteins, and thereby recruited to the viral RNA genome, where it introduces Xm modifications that protect against degradation by host nucleases (94,95). Another recently identified 2′-*O*-ribose RNA MTase is TRMT13, which introduces Xm at position 4 in several different tRNA, but also plays a role in transcriptional regulation (89).

### Other RNA MTases

In addition to m^6^A and m^5^C, a number of other abundant nucleobase methylations exist (Figure 4B), particularly in tRNA, but also in rRNA. Most of the corresponding enzymes have now been identified as 7BS MTases (Figure 3B and Supplementary Table S2). These include MTases generating m^1^A (TRMT61A/B, RRP8) (96–98), 1,*N*^6^-dimethyladenosine (m^6^_2_A; DIMT, TFB1M) (99,100), 3-methylcytosine (m^3^C; METTL2A/B, METTL6 and METTL8) (101), 7-methylguanosine (m^7^G; METTL1, RNMT, BUD23) (100,102,103), *N*^2^-methylguanosine (m_2_G; TRMT11, THUMPD2, THUMPD3) (104,105) and 5-methyluracil (m^5^U; TRMT2A/B) (106,107). The first human m^5^U- and m^2^G-installing 7BS MTases were discovered recently. TRMT2B introduces m^5^U on both rRNA and tRNA in mitochondria (107), whereas TRMT2A installs m^5^U on cytosolic tRNAs (106). TRMT11 and THUMPD3 introduces m^2^G at position 6 and 10, respectively, of tRNAs, whereas THUMPD2 is responsible for generating a single m^2^G modification found in the spliceosomal U6 snRNA (104,105).

The anticodon loop of some tRNAs is modified by bulky adducts – so-called hypermodifications, which are formed through several enzymatic steps, including methylations. For example, ALKBH8 mediates the final methylation step in the formation of methoxycarbonylmethyl-uridine (mcm^5^U) modification on uridines in the wobble position of some tRNAs (Figure 4B) (108–110). Also, two 7BS MTases methylate the 5′ phosphate groups of various RNAs; MePCE (methylphosphate capping enzyme; aka BCDIN3) targets the small nuclear 7SK RNA (111), whereas its paralogue BCDIN3D targets tRNA-His, and possibly also miRNAs (112,113).

## Small molecule (SM) MTases

The 7BS SM MTases constitute a rather diverse group where only a few of the MTases show substantial similarity to each other (Figure 3C). Collectively, the SM MTases target a wide range of small molecules, i.e. neurotransmitters, metabolites and toxic substances. Less attention will be given to this MTase group in this review, as the majority of the SM MTases were discovered > 20 years ago, and these enzymes have been well covered by several excellent review articles (114–119). However, some recent discoveries will be highlighted.

### Neurotransmitter MTases

Several of the SM MTases target key neurotransmitters, playing a role both in their synthesis and in their clearance and/or inactivation. Accordingly, many gene variants of these MTases have been implicated in various brain-related diseases, and studied extensively (115). For example, acetylserotonin *O*-methyltransferase (ASMT) and phenylethanolamine N-methyltransferase (PNMT) catalyse the final steps in the synthesis of melatonin and epinephrine, respectively (115,118), whereas catechol-O-methyltransferase (COMT) is involved in the inactivation of the catecholamine neurotransmitters epinephrine, norepinephrine and dopamine (115). Other neurotransmitter MTases are indolethylamine N-methyltransferase (INMT) and histamine N-methyltransferase (HNMT), which methylate tryptamine and histamine, respectively (115).

### Metabolite MTases

SM MTases also methylate several key metabolites. The last step in creatine synthesis is mediated by guanidinoacetate *N*-methyltransferase (GAMT) (114), and two universally conserved Coenzyme Q (CoQ) MTases, COQ3 and COQ5, catalyse important steps in CoQ synthesis (119). In case of the glycine N-methyltransferase (GNMT) reaction, the product sarcosine (aka N-methylglycine) serves no apparent biological function. Instead, the primary role of GNMT is to convert AdoMet to AdoHcy and thereby regulate the cellular AdoMet levels (116). This occurs primarily in the liver, where GNMT is highly abundant (1–3% of cytosolic protein), and notably, GNMT activity is regulated by folate, the key methyl carrier of the closely connected ‘one carbon metabolism’ (116). Nicotinamide is a form of vitamin B3, and nicotinamide N-methyltransferase (NNMT) plays a key function in clearing excess B3, but has also been implicated in metabolic regulation (117). Finally, thiopurine MTase (TPMT) has been extensively studied for its role in the metabolism of the cancer drug 6-mercaptopurine, but its endogenous substrate has remained elusive (115). However, TPMT was recently found to catalyse a key step in the formation of urothione, an excreted catabolite of molybdopterin (120). Through its ability to bind molybdenum, molybdopterin acts as a cofactor for molybdenum-dependent enzymes.

### Detoxifying MTases

Several MTases play important roles in the protection against harmful agents of both endogenous and exogenous origin. Arsenic is an important environmental toxicant found in the earth's crust, and arsenite(III) methyltransferase (AS3MT) plays, together with redox-enzymes, an important role in its clearance through conversion to methylated species, such as dimethylarsinate(V) (121). The dipeptide carnosine (β-alanine-histidine) is generated in high amounts in several tissues, including brain and muscle, and can act as an antioxidant, metal chelator, and pH buffer (122). Carnosine MTase (CARNMT1) converts carnosine to a more stable, N^1^-histidine methylated form, denoted anserine (123), and, recently, CARNMT1 was also found to generate 1MH in proteins (60). Thus, CARNMT1 represents the only human 7BS MTase that can be placed in more than one of three substrate-based groups, and future research with likely elucidate the relative contributions of its SM and protein MTase activities to its biological function. Several years ago, a membrane associated thiol MTase (TMT) activity was discovered that methylated thiol-containing compounds, such as hydrogen sulphide and various drugs, but the responsible MTase(s) have remained unknown. However, the two related MTases METTL7A and METTL7B were recently identified as the responsible MTases and therefore renamed TMT1A and TMT1B, respectively (124,125).

## Uncharacterized MTases

Despite substantial recent progress, 20 of the human 7BS MTases still remain uncharacterized, and these have here been placed in two categories. The 11 MTases whose type of substrate (protein, DNA/RNA or SM) may be inferred from sequence similarity to already characterized MTases have been denoted as ‘tentative’, and the remaining ones as ‘orphan’ MTases. However, it should be noted that several predicted 7BS MTases later have been found to be devoid of MTase activity and have other functions. One prominent example is SAMTOR, an AdoMet-binding 7BS protein without detected MTase activity. SAMTOR regulates mRNA translation in response to AdoMet (aka SAM) and methionine levels as part of the mTOR complex 1 (mTORC1) pathway, but was initially erroneously annotated as a putative orthologue of the yeast rRNA MTase BMT2 (126). Interestingly, the putative *Drosophila* orthologue of CARNMT1, which is a *N*^1^-histidine MTase in humans, was very recently also found to act as AdoMet-sensor in the mTORC1 pathway (127). This indicates that putatively orthologous MTases may have completely different functions in different organisms, further underscoring the challenge of predicting MTases and their function, and the importance of experimental studies.

### Tentative (protein, DNA/RNA or SM) MTases

PCMTD1 and PCMTD2 are paralogues of the protein repair enzyme PCMT1, and are therefore likely involved in protein modification or repair, although one recent study failed to detect any l-isoaspartate specific protein MTase activity of PCMTD1 (128). Similarly, several of the tentative RNA MTases may be predicted to generate specific methylated RNA bases, based on the product specificity of their respective human paralogues or putative yeast orthologues. Among these are TRMT1L, a paralogue of TRMT1, which generates *N*^2^-dimethylguanosine (m^2^_2_G) in tRNA, as well as NSUN7 (m^5^C; paralogue of NSUN1-6), TF2B2M (m^6^_2_A; paralogue of TF2B1M and DIMT1), FBLL1 (Xm; paralogue of FBL), TRMT44 (Xm; putative orthologue of *S. cerevisiae* Trm44) and LCMT2/TYW4 (wybutosine; putative orthologue of *S. cerevisiae* Tyw4). Finally, COMTD1 and ASMTL display substantial sequence similarity to the SM MTases COMT and ASMT, respectively. Recently, a putatively inactivating mutation in the COMTD1 from chicken was demonstrated to cause defective synthesis of the yellow/red pigment pheomelanin, suggesting that COMTD1 plays a role in its synthesis (129). Interestingly, ASMTL contains, in addition to its MTase domain, also a pyrophosphatase domain, and a corresponding *in vitro* activity was demonstrated on the nucleotides UTP and dTTP, as well as some of their methylated derivatives (130). Thus, it may be speculated that ASMTL plays a role in nucleotide metabolism.

### Orphan MTases

The function of the orphan MTases is largely elusive, but clues on substrates or relevant processes exist for some of them. An MTase domain is found as part of the multifunctional fatty acid synthase (FASN) protein, suggesting a role for the corresponding MTase in modifying fatty acids or regulating their synthesis. Loss of METTL17 leads to reduced levels of specific methylations in rRNA (131), possibly suggesting a role in RNA modification, but this MTase has also been implicated in hormone-mediated gene regulation (132).

## Phylogenetic distribution of 7BS MTases

Several of the human 7BS MTases are highly conserved throughout evolution. This may be illustrated by a comparison with the yeast *S. cerevisiae*, which has 56 7BS MTases. Of these, 39 have putative or established human orthologues, with an additional 9 showing similarity to a specific human 7BS group (based on information from ref. (1)). Conversely, the majority of the human 7BS MTases show similarity to a specific yeast protein, and the remaining MTases typically represent functions/processes that are absent in yeast, e.g. DNA methylation (DNMTs), repair of damage in long-lived proteins (PCMT1), and neurotransmitter synthesis and turnover (e.g. COMT and PNMT). Also, the number of 7BS MTases is considerably higher in humans than in simple model organisms such as yeast, and this appears to have been driven, throughout evolution, mainly by gene duplication events yielding paralogous MTases with similar functions, although new MTases/families have also arisen.

## 7BS MTase-mediated methylations: dynamic regulators—or nuts and bolts?

MTase-mediated methylations on DNA, RNA and protein are important regulators of a myriad of cellular and physiological processes. Epigenetic methylations on DNA and histone proteins, as well as m^6^A modification of mRNA, are prime examples of this. Such methylations are often of low occupancy and highly dynamic, as they can be reversed by dedicated demethylases. Moreover, their effects are typically mediated by proteins carrying reader domains that bind specifically to the methylated molecule.

In contrast, many of the macromolecular methylations introduced by the 7BS MTases appear to be rather static and constitutive. In particular, this seems to be the case for several methylations on rRNA/tRNA and on lysines in non-histone proteins. However, it should be mentioned that in many cases the degree of methylation dynamics remains to be investigated in detail, e.g. under different cellular or physiological states, and that some tRNA methylations have been shown to be dynamic, reversible and to play regulatory roles (133,134). For the static methylations, no demethylases or reader proteins have been identified, and the substrates are typically in a fully methylated state. Thus, such methylations may be viewed as macromolecular editing events that expand the repertoire of biological building blocks, rather than as regulatory signals. In other words, whereas the dynamic methylations represent regulators or switches, the static methylations may be viewed as ‘nuts and bolts’ that improve macromolecular function. However, it should be noted that, for a methylation event to serve a regulatory function, the existence of a demethylase is not a prerequisite. If the methylated molecule has a rapid turnover, dynamic regulation of methylation may be achieved through modulating the amount or activity of the corresponding MTase.

Many diseases, such as cancer, are caused by defects in the cell's regulatory circuitry. Correspondingly, a substantial portion of biological and biomedical research focuses on how cells are regulated and signals transduced. However, the term ‘regulation’ may actually be somewhat overused within the fields of RNA and protein methylation. Typically, when a physiological or cellular process is negatively affected by ablating a specific MTase, e.g. by gene knock-out (KO), it is often concluded that the process is regulated by the relevant methylation event. However, it should be kept in mind that such experiments merely demonstrate that the MTase is of importance, and that the corresponding methylations may well be ‘nuts and bolts’, rather than actual regulators. Still, the constitutive ‘nuts and bolts’ methylations can also be highly disease relevant, as the majority of them decorate the machinery for protein synthesis, which, for example, is highly important for cancer cell growth. Indeed, it was recently found that (apparently) constitutive 7BS MTase mediated methylations on rRNA and the translation factors eEF1A and eEF2 promote tumor formation and cancer cell growth (45,71,135).

## Conflicting substrate assignments and promiscuous MTases

Enzymes are generally highly specific catalysts of biochemical reactions. Still, they often have promiscuous activities, targeting substrates other than the biologically relevant ones, but with lower efficiency. This has proven a great advantage for enzyme evolution, where such promiscuous activities have been leveraged to evolve new enzymes that catalyse desired reactions (136). On the other hand, such promiscuous activities may give challenges when attempting to correctly assign the biologically relevant substrate to an uncharacterized enzyme, such as an MTase. Indeed, some of the mammalian 7BS MTases have, through different studies, been reported to target substrates from more than one of the three substrate groups, and several of the corresponding MTase-substrate assignments are controversial. It may therefore be relevant to ask whether such MTases really have multiple ‘true’ substrates, or whether some of the reported targets reflect promiscuous activities that are not biologically relevant.

One recent example is the reported presence of m^6^A as an epigenetic modification in mammalian DNA (see also the above section on ‘*N*^6^-adenosine MTases’), whose existence still remains controversial, with the implicated MTases also mediating other methylation reactions. It was noted early on that several MTases that target the *N*^6^-position of adenines in DNA share a so-called ‘DPPY’ (consensus D/E-P-P-Y/F) sequence at the position later denoted ‘Motif Post II’ (see above) (137). Based on this, two DPPY-containing human 7BS MTases were given the gene symbols N6AMT1 and N6AMT2 (*N*^6^-adenosine MTases 1 and 2), suggesting a role in m^6^A formation. However, it has later been demonstrated that DPPY motifs are found also in non-DNA MTases. Notably, N6AMT2 has now been firmly established as the long-sought KMT targeting Lys-79 in eEF1A, and consequently renamed eEFA1A-KMT1 (gene: *EEF1AKMT1*). N6AMT1 has been reported to introduce m^6^A in mammalian DNA (138), but other studies failed to reproduce these findings (77). This MTase was also reported as a histone KMT (139), but has been most convincingly established as a Gln MTase targeting translation release factors (62,140). Also the DPPY-containing MTase METTL4, which is a close relative of the RNA MTase METTL3, shows apparent substrate promiscuity. It was reported to introduce m^6^A in both nuclear and mitochondrial DNA (141,142), but has also been identified as the elusive MTase introducing m^6^A at a specific position in U2 small nuclear RNA (snRNA) (74). Furthermore, the much-studied MTase METTL3/METTL14, which is responsible for pervasive m^6^A in mRNA, also shows robust activity on DNA *in vitro*, but the significance of this remains unclear (143). Clearly, for several of the human 7BS MTases, it will be important to clarify whether their reported promiscuities are real and biologically significant.

## Studying 7BS MTases

The vast majority of the human 7BS MTases were first identified as theoretical proteins, i.e. putative MTases, and subsequently characterized through experimental studies. The MTases have been investigated both regarding the specific biochemical reactions they catalyse and their functional importance in cells and organisms. Results from such different lines of research are then typically combined, giving novel insights and hypotheses for further research. Obviously, the challenges faced when studying different MTases can vary greatly. Some MTases appear to have only a single substrate, whereas others have a multitude of substrates, making it more complicated to assess the functional significance of the MTase and the corresponding methylations. Also, some MTases mediate the complete methylation of their substrates, while others typically catalyse low-occupancy methylations, the relevance of which may be harder to entangle. To avoid erroneous or irrelevant assignment of MTase substrates, it is therefore critical that *in vitro* and *in vivo*/cellular approaches are combined, and that orthogonal methods are used. In the case of lysine specific MTases, this has been addressed in an excellent article by Kudiputhi and Jeltsch (144). Here, I will describe some key experimental strategies for MTase studies, and some major challenges will be addressed.

### *In vitro* studies

The measurement of MTase activity in a test tube represents a cornerstone of MTase research. It has been used extensively for uncovering the biochemical activities of MTases, but also for addressing molecular mechanisms through structure-function studies, and when developing inhibitors of medically relevant MTases. In principle, an MTase reaction requires only three components, i.e. substrate, MTase and the methyl donor AdoMet. For most 7BS MTases, *in vitro* activity can be observed with only these three components.

For *in vitro* MTase experiments, an affinity-tagged, *E. coli*-expressed, recombinant MTase is usually used and gives robust enzymatic activity, but there may also be additional requirements, e.g. for accessory proteins or specific post-translational modifications on the MTase. In such cases, MTase activity may be obtained by co-expressing accessory proteins with the MTase, or by using insect cells or human cell lines as the expression host. Similar issues exist also for the macromolecular protein and DNA/RNA substrates. Here, activity can often be observed on simple substrates such as peptides, oligonucleotides, recombinant proteins or *in vitro*-transcribed RNAs, but there are exceptions, and even instances where all efforts to detect *in vitro* MTase activity have failed. In such cases, the substrate may need to be in the context of a particular macromolecular complex or folding/assembly intermediate, to have additional (post-translational or post-transcriptional) modifications, or to be bound to a specific small molecule.

It should also be noted that when using recombinant *E. co*li-expressed MTases, activities of co-purifying endogenous *E. coli* MTases may mistakenly be interpreted to represent the recombinant MTases. Similar problems can be experienced with other expression/purification systems, for example when using anti-FLAG antibody to pull down FLAG-epitope tagged MTases from human cell extracts. The anti-FLAG antibodies will also pull down the highly active protein MTase PRMT5, causing potential misinterpretation of the results (145). To avoid these caveats, it is essential to use a catalytically inactive mutant MTase as a control, and to also validate the results using orthogonal approaches.

AdoMet containing a radiolabel, typically tritium ([^3^H]), in the transferred methyl group has been a key research tool since the early days of MTase research, allowing highly sensitive and specific detection of the methylation products. It is still used extensively, but has now been complemented by non-radioactive methods. Foremost, developments within MS have facilitated specific and sensitive detection of non-radioactive methylation products in complex mixtures (see below). Here, tritiated AdoMet may be replaced by its deuteriated counterpart, thus allowing specific detection of the heavy methylation product by MS; this may be particularly useful if the substrate is present in a mixture (e.g. a cell extract) that already contains the product of methylation (45,71). Also, the use of deuteriated AdoMet will allow distinguishing between MTase-mediated methylations and spurious methylations resulting from methanol exposure during sample preparation, which is a problem when studying protein methylation (146). Besides the MTase assays that measure the actual product of methylation, indirect assays have been developed that detect the formation of AdoHcy. Such assays, e.g. the MTase-Glo™ assay, couple the formation of AdoHcy, through enzymatic conversions, to the generation of a readily detectable signal, e.g. luminescence (147). Thus, these methods can be readily adapted to a high-throughput format, and are therefore widely used for drug screens. However, they are vulnerable to artefacts, and should not be used for initial characterization of MTase activity, but only to assess activities that have already been firmly established using more rigorous methods.

### Assessing methylation *in vivo*

Besides establishing methylation of a specific substrate by a given MTase *in vitro*, it is of course equally important to determine whether, and to what extent, the corresponding methylation reaction occurs physiologically. This is typically addressed by measuring both the (unmethylated) substrate and the (methylated) product, and comparing their levels between MTase deficient, (typically KO) cells (or tissues) and wild-type (WT) cells, sometimes also using cell lines that overexpress the MTase. If the level of the methylated product is higher in WT cells than in KO cells, whereas the opposite is observed for the unmethylated substrate, this is a strong indication that the MTase catalyses the corresponding reaction also *in vivo*. Such experiments have been greatly facilitated by the advent of CRISPR-based gene KO, which is the method of choice when the MTase is non-essential for cellular/organismal survival. For essential MTases, partial knock-down, e.g. through siRNA-mediated interference, may be used instead.

Many 7BS MTases, especially those that mediate RNA and protein lysine methylation, share the following features: (a) they act on a single substrate or a few related substrates, (b) the substrate's methylation level is typically high (close to 100%) and (c) no redundant MTase exists that mediates the same reaction. In these cases, experiments that compare MTase KO and WT cells typically give black-and-white results that firmly establish the *in vivo* activity of the MTase, i.e. the KO cells contain exclusively the non-methylated form of the substrate, whereas the methylated form is predominant in the WT cells. In contrast, the picture may be much more complex and challenging to interpret for redundant MTases and broad-specificity MTases that mediate low occupancy methylations, as well as for small molecule MTases whose substrates and products can be metabolized by a number of enzymes.

### Studying methylation by mass spectrometry

MS based methods represent a cornerstone for *in vivo* detection of MTase products. In the case of proteins, they are typically digested to peptides that are subjected to electrospray LC–MS/MS, allowing both their sequencing and quantitation. However, it should be noted that false positives can be a problem in large-scale, MS-based identification of protein methylation (146). Similarly, RNase-generated RNA fragments can be sequenced and thus investigated for their methylation status by using MALDI (matrix-assisted laser desorption/ionization)-MS (148).

Obviously, small-molecule LC–MS(/MS) is used extensively for assessing the substrates and products of SM MTases, but it can also be used to assess macromolecular methylation. Nucleic acids are then enzymatically digested to their constituent nucleosides, whereas proteins are degraded to amino acids through acid hydrolysis (57,149). The overall content of a given methylated residue(s) in the sample is then measured by the LC–MS(/MS) analysis, which also allows the distinction, through different LC retention times, between isomers, which are not readily distinguishable in standard MS, e.g. m^1^A versus m^6^A, or 1MH versus 3MH.

### Detection of macromolecular methylation by antibodies

Antibodies developed to specifically recognize methylated macromolecules are useful tools for studying *in vivo* methylation, with the caveat that even the best antibodies may have some level of non-specific signal. Some antibodies recognize a specific type of methylation, such as m^6^A in RNA and methylated Lys in protein, regardless of its sequence and structural context. These ‘pan-antibodies’ have been particularly useful for specific enrichment of methylated peptides or RNA fragments prior to subsequent analysis such as MS or RNA sequencing, but the caveat exists that many of the alleged pan-antibodies actually do show pronounced sequence preferences.

Also, a number of antibodies have been generated that recognize individual methylation sites in proteins, and are typically used to quantitate and localize specific methylated proteins by western blotting and immunofluorescence microscopy, respectively. However, it is key to properly validate such site- and methylation-specific antibodies, assuring that the observed signal really is due to recognition of the desired target sequence. This may be done by using MTase KO cells; if recognition is specific, the observed methylation signal should expectedly be absent in cells that lack the responsible MTase.

### High-throughput sequencing-based methods for assessing DNA and mRNA methylation

Several of the methods used to assess methylation of nucleic acids take advantage of alterations in chemical properties caused by methylation. A classic example is the use of bisulfite sequencing to assess m^5^C in DNA and RNA; bisulfite treatment causes deamination of cytosine to uracil, but m^5^C is refractory to such treatment, and the methylated residues are identified by sequencing (150). More recently, methods have been developed that instead of bisulfite treatment use enzymatic deamination, mediated by the cytosine deaminase APOBEC3A (151,152). Similarly, 2′-*O*-methylation of the RNA ribose moiety renders RNA less susceptible to alkaline hydrolysis, and methylation sites can thus be detected as ‘missed cleavage sites’ when sequencing the fragments obtained by alkaline treatment of RNA (153).

Other methods use methylation specific antibodies or affinity-tagged MTases to specifically pull-down methylated RNA fragments that are subsequently sequenced, often using crosslinking agents that strengthen the interactions. For example, in the ‘m^6^A individual-nucleotide-resolution cross-linking and immunoprecipitation’ (miCLIP) method, cellular mRNAs are fragmented and subjected to immunoprecipitation with m^6^A-specific antibodies followed by UV-crosslinking (154). The RNA fragments are then reverse transcribed into cDNA, and during this process, specific mutations are introduced at the methylation sites due to the presence of antibody remnants. The mutations, which are uncovered by subsequent cDNA sequencing, are then used to specifically locate the methylation sites.

### Using AdoMet analogues for MTase studies

Many DNA/RNA, protein and SM MTases are able to use as co-factors AdoMet analogues where the methyl group has been replaced by a bulkier alkyl group, which is transferred to the relevant substrate during the MTase reaction (155–157). Furthermore, if the alkyl group contains an alkyne moiety, this allows for highly specific conjugation of a desired entity, such as a fluorescent label or biotin to the MTases substrate. This approach has been used for specific labelling of desired nucleic acid sequences and subspecies (158–160), but has been particularly useful for identifying substrates of protein MTases, including several human members of the 7BS family (47,57,161). Typically, a cell extract is then incubated with the MTase and an azide-containing AdoMet analogue, leading to the transfer of the azide-containing moiety to the relevant substrate. Using click chemistry, a biotin moiety is specifically added to the substrate proteins, allowing their isolation using streptavidin, and identification by MS. In such AdoMet analogues, the S-atom is often replaced by selenium to increase the stability, and one of these, ProSeAM (propargylic Se-adenosyl-L-selenomethionine), has been particularly widely used, as it is accepted by a number of different MTases (162,163). Some MTases are unable to efficiently catalyse transferase reactions using such AdoMet analogues, but this may be amended by modifying the MTase active site (164,165).

Another expansion of this strategy, which has particularly been explored and developed for certain SET-domain MTases, is the coordinated structure-based optimization of the MTase substrate binding site (by amino acid replacement) and the AdoMet analogue, yielding highly specific and efficient reaction (166). Such ‘bio-orthogonal’ MTase-cofactor pairs enable unique labelling of the substrates of a single MTase. Cells are normally impermeable to AdoMet and its analogues, but *in vivo* labelling can be achieved by feeding cells methionine derivatives, which are converted to the corresponding AdoMet analogues inside cells (165,167).

### Functional effects of methylation

When it has been established, both *in vitro* and in cells, that a given MTase targets one or more substrates, it is of great interest to establish the functional consequences, if any, of this methylation event(s). This may be challenging, but generally less so for MTases that only target a single substrate. The functional effect(s) of a given methylation event may be studied at several levels: (i) by assessing the biochemical properties of the targeted biomolecule, (ii) by studying the cellular process in which the biomolecule is involved and (iii) through studying phenotypes of organisms that lack the responsible MTase. One recent example of such multilevel studies of MTase function is that of METTL13, a dual MTase which targets two residues in the universally conserved translation elongation factor and GTPase eEF1A (44,45). Here, it was demonstrated that methylation of eEF1A stimulated GTPase activity *in vitro*, and was required for efficient mRNA translation and growth of cancer cells. Accordingly, mice devoid of METTL13 showed a reduced growth of certain tumours (45). Clearly, to fully understand the function of an MTase, it is key to carry out investigations at several levels.

## 7BS MTases and cancer

Collectively, the human 7BS MTases have been associated a wide range of diseases (115). One prominent example is COMT, which is involved in the inactivation of several key neurotransmitters (168). COMT has been implicated in a number of neurophysiological disorders such as schizophrenia and depression, and, notably, one single common COMT polymorphism, Val158Met, has been subject of intense study (>1000 research articles)(115). However, the general topic of MTases and disease is obviously too large to be covered here; thus the discussion will be limited to some key points related to cancer.

**Figure 5.**
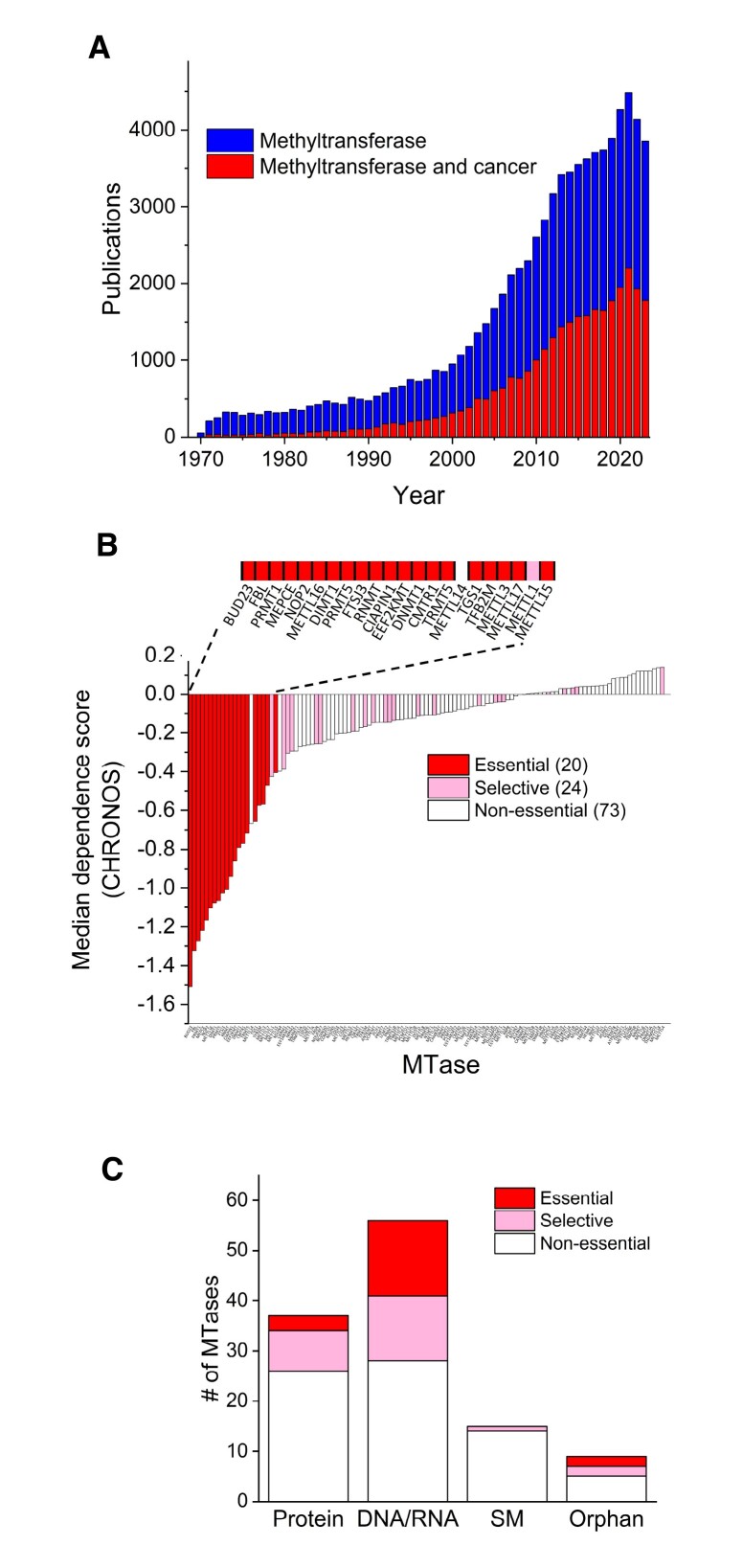
7BS MTases and cancer. (**A**) Timeline for the number of publications that contain either ‘methyltransferase’ and ‘methyltransferase AND cancer’ in their title or abstract (from PubMed). (**B**) Essentiality of 7BS MTases in human cancer cell lines assessed by using DepMap. The DepMap resource (https://depmap.org/) contains data from experiments where the essentiality of human genes was assessed by systematically knocking them out (using CRISPR) in a panel of ∼1100 human cancer cell lines, and then measuring cell viability. Based on these results, genes were categorized as either essential, non-essential, or selective, where the latter represents genes that are essential only in a subset of the cell lines. Also, the degree of essentiality was calculated using a CHRONOS score, where -1 and 0 represents the average of essential and non-essential genes, respectively. 117 of the 120 7BS MTases were analysed (data for ASMT, ASMTL, FBLL1 were not available). Supplementary Figure S1 shows a zoomed-in, higher resolution version of this panel, allowing viewing of the results for each individual MTase. (**C**) Groupwise presentation of the essentiality of the 7BS MTases.

To illustrate the cancer relevance of MTases, a PubMed search using the queries ‘methyltransferase’ and ‘methyltransferase AND cancer’ was performed. The results showed that MTase research, as well as the proportion of cancer-related articles, have been steadily increasing, with about half of the articles from recent years having (per the above search criterion) a link to cancer (Figure 5A). It should, however, be noted that a substantial fraction of the articles can be subscribed to the SET domain enzymes, which are responsible for the bulk of epigenetic histone lysine methylations. Still, numerous 7BS MTases have been implicated in cancer, and several of these have been developed as drug targets. In particular, there has been a focus on targeting MTases introducing epigenetic histone or DNA methylations, such as the 5-cytosine MTase DNMT1, the arginine MTase PRMT5 and the KMT DOT1L (169–171). For these MTases, extensive drug development efforts have been undertaken, resulting in several inhibitors that have reached clinical trials, or are now even approved as cancer drugs.

To generally assess the importance of the 7BS MTases for cancer cell growth, I utilized data from the DepMap portal. This resource contains data from experiments where human genes where individually knocked out by CRISPR technology in ∼1100 cancer cell lines (172). Based on the results, a so-called CHRONOS score has been calculated, where essential and non-essential genes typically yield values of –1 and 0, respectively (173). Furthermore, the individual genes were then categorized as being either essential, non-essential, or selective, where the latter are essential only in a subset of the cell lines, and thus may have potential as drug targets in specific cancers. DepMap data could be retrieved for 117 out of the 120 human 7BS MTases, and of these 20 were essential, 73 non-essential and 24 selective (Figure 5B). Some interesting differences in the DepMap data are observed between the different MTase groups. About half of the DNA/RNA MTases and close to one third of the protein MTases were either essential or selective, whereas only a single SM MTase belonged to either of these categories (Figure 5C). This agrees well with many of the DNA/RNA and protein MTases being highly conserved in evolution and with clearly defined orthologues in simple eukaryotes, reflecting their roles in basic cellular functions. In contrast, the SM MTases are generally less conserved during evolution, reflecting that they are mostly involved in specialized, often tissue-specific, processes unique to multicellular eukaryotes.

The initiation step of mRNA translation is subject to tight regulation, and how cancer cells rewire translational initiation to support their growth is a subject of intense studies (174). In contrast, the elongation step of translation is less regulated and also less studied in the context of cancer. Interestingly, six distinct 7BS MTase activities target the N-terminus and five lysines residues in the essential elongation factor eEF1A, and the 7BS MTase eEF2-KMT (FAM86A) methylates Lys-525 in the other main elongation factor, eEF2. The corresponding methylations appear to have optimizing rather than regulatory roles, as they are mostly of high occupancy and apparently not reversed by demethylases. Still, these MTases may be highly relevant as cancer drug targets, as cancer cells depend on efficient protein synthesis to sustain their growth. Indeed, it was demonstrated that both eEF2-KMT and the eEF1A MTase METTL13 were required for efficient growth of Ras-driven cancer, and that METTL13 depletion rendered the cancer cells hypersensitive towards other cancer drugs (45,135). Interestingly, both METTL13 and two of the other eEF1A-specific MTases, eEF1A-KMT1 and eEF1A-KMT3, are found in the ‘Selective’ category, and, conceivably, highly specific inhibitors directed towards these MTases may find applications as cancer drugs towards certain cancers, and with less side effects than drugs that target essential MTases. Moreover, a large proportion of the 7BS MTases target tRNA or rRNA, and several of these are in the ‘Essential’ or ‘Selective’ categories, and targeting of RNA MTases has recently emerged as a very interesting avenue for cancer treatment (175,176).

Many studies addressing the 7BS MTase function in cancer are excellent and have opened up new avenues regarding cancer treatment. On the other hand, a significant fraction of the studies are still too superficial and yield limited insights. In particular, many of them implicate a given MTase in a certain cancer type, but without paying much or any attention to the actual reaction catalysed by the MTase. Such studies often follow a similar template where the MTase is first found to be highly expressed in a specific cancer type. This is typically followed up by studying the effect of MTase ablation and overexpression on the growth of corresponding cancer cell lines in tissue culture or as mouse xenograft tumours, and, finally, clinical patient data such as on overall survival, is compared for cancer patients who have low versus high levels of the MTase. Although such studies provide some indications for a role for the MTase in cancer, many such studies could benefit from also assessing the actual product of methylation, and further investigate the molecular pathways by which this product influences cancer growth. This would also aid in overcoming the barrier from merely correlative studies to mechanistic understanding.

## The 7BS MTase and METTL gene naming nomenclature

When Petrossian and Clarke's ‘methyltransferasome’ overview was published in 2011 (1), characterized MTases such as PRMTs and SM MTases already carried names/symbols that reflected their biochemical activities. However, the majority of the 7BS MTases existed merely as theoretical open reading frames (ORFs) predicted from genomic and cDNA sequences, and the genes encoding them were denoted by placeholder symbols such as *C14orf138* (‘Chromosome 14 Open Reading Frame 138″) and *FAM173A* (‘family with sequence similarity 173 member A’). While most 7BS MTases with a C#orf placeholder symbol were renamed as METTLs, those with the placeholder symbol FAM# (used to group closely related but uncharacterized paralogs) were not. Thus, the 34 7BS MTases that were once given a METTL name merely represents an arbitrarily and historically selected subset of the 120 human 7BS MTases (Figure 2B). Still, the METTLs are often perceived as a distinct MTase group, and several review articles and omics studies dedicated to the METTLs have been published.

Adding to the picture, several of the METTLs, in particular the lysine-specific MTases (KMTs), have had their HGNC approved nomenclature updated to use more informative symbols. For example, METTL21D is now denoted VCP-KMT (gene name: *VCPKMT*), and METTL19 was renamed TRMT44, being a putative orthologue of the yeast tRNA MTase Trm44. Consequently, only 24 of the human 7BS MTases currently have a METTL-type name as their formal gene symbol. Many of the METTLs have been highly published on, and their symbols have become entrenched in the literature. In such cases (e.g. *METTL1*), it would not be helpful for their approved symbols to be changed, unless the whole community working in a field would strongly support such a change. In some other cases (e.g. *METTL25*), little is yet known about their function, and their METTL symbol will likely be retained, at least until they are better characterized.

## Concluding remarks and future perspectives

Clearly, tremendous progress has in recent years been made on the unravelling of the biochemical activities of the human 7BS MTases. Still, knowledge on the functional roles of the corresponding methylations are in many cases completely lacking, especially for the numerous RNA and protein methylations. Thus, it will be of great interest to investigate how the methylations influence biomolecular structure, function and dynamics, and also to study effects of MTase KO in mouse models. A large proportion of the MTases target components of the cellular machinery for protein synthesis, such as tRNAs, rRNAs, and translation factors, and it will be a challenge for future studies to investigate the extent of crosstalk and co-regulation between such methylations.

## Supplementary Material

gkae816_Supplemental_File
