# Supplementary material for: Closing in on human methylation—the versatile family of seven-β-strand (METTL) methyltransferases
Source: Nucleic Acids Res. 2024 Oct 1;52(19):11423–41. doi: 10.1093/nar/gkae816 (PMC11514484; doi:10.1093/nar/gkae816)

Supplementary information

Closing in on human methylation - the versatile family of seven-β-strand (METTL) methyltransferases

Pål Ø. Falnes

Supplementary Table 1: Human protein (seven-β-strand) MTases

Supplementary Table 2: Human DNA/RNA (seven-β-strand) MTases

Supplementary Table 3: Human small molecule (seven-β-strand) MTases

Supplementary Figure 1: Zoomed-in version of Figure 5B

Supplementary Table 1. Human protein (seven-β-strand) MTases

For a given MTase, the listed reference(s) describes the characterization of the MTase in the indicated year (which is also used in the timeline in Fig. 2A). The information on substrates/products are based on these references, as well as on original articles and reviews referred to in the main paper. For single-substrate MTases, the substrate is usually referred to by its official gene name from the HGNC (Human Gene Nomenclature Committee;https://www.genenames.org/) name; when not, further explanation and official gene names are given below the table. In some cases, the amino acid numbering does not refer to the full-length/precursor version of the substrate protein, but to the mature protein where the initiator methionine or mitochondrial pre-sequence has been cleaved off; this is indicated by # or $ respectively. The MTases have been grouped according to their products, with the two largest subgroups, KMTs and PRMTs, shaded in blue and red respectively. Within each subgroup the MTases are sorted alphabetically on gene name.

UniProt ID	Gene symbol	Alias	Status	UniProt name	Substrate	Product	Year	Ref.
Q96MG8	PCMTD1		Tent	Protein-L-isoaspartate O-MTase domain-containing protein 1	?	?	-----	
Q9NV79	PCMTD2		Tent	Protein-L-isoaspartate O-MTase domain-containing protein 2	?	?	-----	
Q9H1A3	METTL9	DREV	Est	Protein-L-histidine N-pros-methyltransferase	His in HxH motifs	1MH	2021	(1-3)
O95568	METTL18		Est	Histidine protein methyltransferase 1 homolog	H245 in RPL3	3MH	2021	(4,5)
P22061	PCMT1	PIMT	Est	Protein-L-isoaspartate(D-aspartate) O-methyltransferase	IsoAsp in proteins	IsoAsp-me	1987	(6)
Q9BQD7	ANTKMT	FAM173A	Est	Adenine nucleotide translocase lysine N-methyltransferase	K52 in ANT	Kme3	2019	(7)
Q6P4H8	ATPSCKMT	FAM173B	Est	ATP synthase subunit C lysine N-methyltransferase	K43$ in ATPSc	Kme3	2018	(8)
Q7Z624	CAMKMT		Est	Calmodulin-lysine N-methyltransferase	K115# in calmodulin	Kme3	2010	(9)
A8MUP2	CSKMT	METTL12	Est	Citrate synthase-lysine N-methyltransferase	K395 in CS	Kme3	2017	(10,11)
Q8TEK3	DOT1L	KMT4	Est	Histone-lysine N-methyltransferase, H3 lysine-79 specific	K79# in histone H3	Kme1,2,3	2002	(12)
Q8WVE0	EEF1AKMT1	N6AMT2	Est	EEF1A lysine methyltransferase 1	K79 in EEF1A	Kme3	2015	(13)
Q5JPI9	EEF1AKMT2	METTL10	Est	EEF1A lysine methyltransferase 2	K318 in EEF1A	Kme3	2014	(14)
Q96AZ1	EEF1AKMT3	METTL21B, FAM119B	Est	EEF1A lysine methyltransferase 3	K165 in EEF1A	Kme1,2,3	2017	(15,16)
P0DPD7	EEF1AKMT4	ECE2	Est	EEF1A lysine methyltransferase 4	K36 in EEF1A	Kme3	2017	(17)
Q96G04	EEF2KMT	FAM86A	Est	Protein-lysine N-methyltransferase EEF2KMT	K525 in EEF2	Kme3	2014	(18)
Q8IXQ9	ETFBKMT	METTL20	Est	Electron transfer flavoprotein beta subunit lysine methyltransferase	K200 and K202 in ETFB	Kme1,2,3	2014	(19,20)
Q8N6R0	METTL13	EEF1AKNMT, FEAT	Est	eEF1A lysine and N-terminal methyltransferase (eEF1A-KNMT)	N-term. and K55 in EEF1A	Kme2; Nt-me3	2018	(21,22)
Q8WXB1	METTL21A	FAM119A	Est	Protein N-lysine methyltransferase METTL21A	K561 in HSPA1	Kme3	2013	(23,24)

Q5VZV1	METTL21C		Est	Protein-lysine methyltransferase METTL21C	K943 in AARS1	Kme1,2,3	2020	(25)
Q9BUU2	METTL22		Est	Methyltransferase-like protein 22	K135 in KIN	Kme3	2013	(23)
Q9H867	VCPKMT	METTL21D	Est	VCP lysine methyltransferase	K315 in VCP	Kme3	2012	(23,26)
Q86X55	CARM1	PRMT4	Est	Histone-arginine methyltransferase CARM1	Several	MMA, ADMA	1999	(27)
Q86XA0	METTL23		Est	Histone-arginine methyltransferase METTL23	R17# in histone H3	MMA, ADMA	2017	(28)
Q7L592	NDUFAF7		Est	Protein arginine methyltransferase NDUFAF7	Arg85$ in NDUFS2	SDMA	2013	(29)
Q99873	PRMT1		Est	Protein arginine N-methyltransferase 1	Several (RG/RGG motifs)	MMA, ADMA	1996	(30)
P55345	PRMT2		Est	Protein arginine N-methyltransferase 2	Uncertain	MMA, ADMA	2009	(31)
O60678	PRMT3		Est	Protein arginine N-methyltransferase 3	Several	MMA, ADMA	1998	(32,33)
O14744	PRMT5		Est	Protein arginine N-methyltransferase 5	Several	MMA, SDMA	2001	(34)
Q96LA8	PRMT6		Est	Protein arginine N-methyltransferase 6	Histone H3 (R2#) and others	MMA, ADMA	2002	(35)
Q9NVM4	PRMT7		Est	Protein arginine N-methyltransferase 7	RxR motifs	MMA	2004	(36,37)
Q9NR22	PRMT8		Est	Protein arginine N-methyltransferase 8	Several	MMA, ADMA	2005	(38)
Q6P2P2	PRMT9	PRMT10	Est	Protein arginine N-methyltransferase 9	Splicing factors and others	MMA, SDMA	2015	(39)
Q9UIC8	LCMT1	LCMT	Est	Leucine carboxyl methyltransferase 1	L309 (COOH) in PPP2CA	Leu-COO-me	1999	(40)
Q9BV86	NTMT1	METTL11A, NRMT1	Est	N-terminal Xaa-Pro-Lys N-methyltransferase 1	N-terminal X-Pro-Lys	Nt-me1,2,3	2010	(41,42)
Q5VVY1	NTMT2	METTL11B, NRMT2	Est	N-terminal Xaa-Pro-Lys N-methyltransferase 2	N-terminal X-Pro-Lys	Nt-me3	2013	(43)
Q9Y5N5	N6AMT1	HEMK2, KMT9	Est	Methyltransferase N6AMT1	Gln-185 in ETF1	Qme1	2008	(44)
Q9Y5R4	HEMK1	HEMK	Est	MTRF1L release factor glutamine methyltransferase	Gln-252 in MTRF1L	Qme1	2008	(45)

Targets – abbreviations and additional information: ANT, three similar genes ANT1-3, ATPSc, ATP synthase C subunit, the three genes ATP5MC1-3 encode identical mature proteins; EEF1A, two similar paralogous genes EEF1A1 and EEF1A2; Est, established; histone H3, several identical genes, e.g. H3C1; HSPA1, the corresponding Lys residue in other Hsp70/HSPA proteins (e.g. HSPA5 and HSPA8) is also methylated; Tent, tentative

Products – abbreviations: 1MH, 1-methylhistidine; 3MH, 3-methylhistidine; IsoAsp, isoaspartate; IsoAsp-me, L-isoaspartate alpha-methyl ester; Kme1, monomethylated lysine; Kme2, dimethylated lysine; Kme3, trimethylated lysine; Nt-me1, monomethylated N-terminal amino group; Nt-me2, dimethylated N-terminal amino group; Nt-me3, trimethylated N-terminal amino group; MMA, monomethylarginine; ADMA, asymmetric dimethylarginine; SDMA, symmetric dimethylarginine; Leu-COO-me; C-terminal leucine methyl ester; Qme1, methylated glutamine.

References for Supplementary Table 1

1. Daitoku,H., Someya,M., Kako,K., Hayashi,T., Tajima,T., Haruki,H., Sekiguchi,N., Uetake,T., Akimoto,Y. and Fukamizu,A. (2021) siRNA screening identifies METTL9 as a histidine Npi-methyltransferase that targets the proinflammatory protein S100A9. *J.Biol.Chem.*, **297**, 101230.
2. Davydova,E., Shimazu,T., Schuhmacher,M.K., Jakobsson,M.E., Willemen,H.L.D.M., Liu,T., Moen,A., Ho,A.Y.Y., Malecki,J., Schroer,L., Pinto,R. *et al.* (2021) The methyltransferase METTL9 mediates pervasive 1-methylhistidine modification in mammalian proteomes. *Nat.Commun.*, **12**, 891.

3. Lv,M., Cao,D., Zhang,L., Hu,C., Li,S., Zhang,P., Zhu,L., Yi,X., Li,C., Yang,A., Yang,Z. *et al.* (2021) METTL9 mediated N1-histidine methylation of zinc transporters is required for tumor growth. *Protein Cell*, **12**, 965-970.
4. Malecki,J.M., Odonohue,M.F., Kim,Y., Jakobsson,M.E., Gessa,L., Pinto,R., Wu,J., Davydova,E., Moen,A., Olsen,J.V., Thiede,B. *et al.* (2021) Human METTL18 is a histidine-specific methyltransferase that targets RPL3 and affects ribosome biogenesis and function. *Nucleic Acids Res.*, **49**, 3185-3203.
5. Matsuura-Suzuki,E., Shimazu,T., Takahashi,M., Kotoshiba,K., Suzuki,T., Kashiwagi,K., Sohtome,Y., Akakabe,M., Sodeoka,M., Dohmae,N., Ito,T. *et al.* (2022) METTL18-mediated histidine methylation of RPL3 modulates translation elongation for proteostasis maintenance. *Elife.*, **11**, e72780.
6. McFadden,P.N. and Clarke,S. (1987) Conversion of isoaspartyl peptides to normal peptides: implications for the cellular repair of damaged proteins. *Proc.Natl.Acad.Sci.U.S.A*, **84**, 2595-2599.
7. Malecki,J.M., Willemen,H.L.D.M., Pinto,R., Ho,A.Y.Y., Moen,A., Eijkelkamp,N. and Falnes,P.O. (2019) Human FAM173A is a mitochondrial lysine-specific methyltransferase that targets adenine nucleotide translocase and affects mitochondrial respiration. *J Biol.Chem.*, **294**, 11654-11664.
8. Malecki,J.M., Willemen,H.L.D.M., Pinto,R., Ho,A.Y.Y., Moen,A., Kjonstad,I.F., Burgering,B.M.T., Zwartkruis,F., Eijkelkamp,N. and Falnes,P.O. (2018) Lysine methylation by the mitochondrial methyltransferase FAM173B optimizes the function of mitochondrial ATP synthase. *J Biol.Chem.*, **294**, 1128-1141.
9. Magnani,R., Dirk,L.M., Trievel,R.C. and Houtz,R.L. (2010) Calmodulin methyltransferase is an evolutionarily conserved enzyme that trimethylates Lys-115 in calmodulin. *Nat.Commun.*, **1**, 43.
10. Malecki,J., Jakobsson,M.E., Ho,A.Y.Y., Moen,A., Rustan,A.C. and Falnes,P.O. (2017) Uncovering human METTL12 as a mitochondrial methyltransferase that modulates citrate synthase activity through metabolite-sensitive lysine methylation. *J Biol.Chem.*, **292**, 17950-17962.
11. Rhein,V.F., Carroll,J., Ding,S., Fearnley,I.M. and Walker,J.E. (2017) Human METTL12 is a mitochondrial methyltransferase that modifies citrate synthase. *FEBS Lett.*, **591**, 1641-1652.
12. Feng,Q., Wang,H., Ng,H.H., Erdjument-Bromage,H., Tempst,P., Struhl,K. and Zhang,Y. (2002) Methylation of H3-lysine 79 is mediated by a new family of HMTases without a SET domain. *Curr.Biol.*, **12**, 1052-1058.
13. Hamey,J.J., Winter,D.L., Yagoub,D., Overall,C.M., Hart-Smith,G. and Wilkins,M.R. (2015) Novel N-terminal and lysine methyltransferases that target translation elongation factor 1A in yeast and human. *Mol.Cell Proteomics.*, **15**, 164-176.
14. Shimazu,T., Barjau,J., Sohtome,Y., Sodeoka,M. and Shinkai,Y. (2014) Selenium-based S-adenosylmethionine analog reveals the mammalian seven-beta-strand methyltransferase METTL10 to be an EF1A1 lysine methyltransferase. *PLoS.ONE.*, **9**, e105394.
15. Hamey,J.J., Wienert,B., Quinlan,K.G.R. and Wilkins,M.R. (2017) METTL21B Is a Novel Human Lysine Methyltransferase of Translation Elongation Factor 1A: Discovery by CRISPR/Cas9 Knockout. *Mol.Cell Proteomics.*, **16**, 2229-2242.
16. Malecki,J., Aileni,V.K., Ho,A.Y., Schwarz,J., Moen,A., Sorensen,V., Nilges,B.S., Jakobsson,M.E., Leidel,S.A. and Falnes,P.O. (2017) The novel lysine specific methyltransferase METTL21B affects mRNA translation through inducible and dynamic methylation of Lys-165 in human eukaryotic elongation factor 1 alpha (eEF1A). *Nucleic Acids Res.*, **45**, 4370-4389.
17. Jakobsson,M.E., Malecki,J., Nilges,B.S., Moen,A., Leidel,S.A. and Falnes,P.O. (2017) Methylation of human eukaryotic elongation factor alpha (eEF1A) by a member of a novel protein lysine methyltransferase family modulates mRNA translation. *Nucleic Acids Res.*, **45**, 8239-8254.
18. Davydova,E., Ho,A.Y., Malecki,J., Moen,A., Enserink,J.M., Jakobsson,M.E., Loenarz,C. and Falnes,P.O. (2014) Identification and characterization of a novel evolutionarily conserved lysine-specific methyltransferase targeting eukaryotic translation elongation factor 2 (eEF2). *J.Biol.Chem.*, **289**, 30499-30510.
19. Malecki,J., Ho,A.Y., Moen,A., Dahl,H.A. and Falnes,P.O. (2015) Human METTL20 is a Mitochondrial Lysine Methyltransferase that Targets the Beta Subunit of Electron Transfer Flavoprotein (ETFbeta) and Modulates Its Activity. *J.Biol.Chem.*, **290**, 423-434.

20. Rhein,V.F., Carroll,J., He,J., Ding,S., Fearnley,I.M. and Walker,J.E. (2014) Human METTL20 methylates lysine residues adjacent to the recognition loop of the electron transfer flavoprotein in mitochondria. *J.Biol.Chem.*, **289**, 24640-24651.
21. Jakobsson,M.E., Malecki,J.M., Halabelian,L., Nilges,B.S., Pinto,R., Kudithipudi,S., Munk,S., Davydova,E., Zuhairi,F.R., Arrowsmith,C.H., Jeltsch,A. *et al.* (2018) The dual methyltransferase METTL13 targets N terminus and Lys55 of eEF1A and modulates codon-specific translation rates. *Nat.Commun.*, **9**, 3411.
22. Liu,S., Hausmann,S., Carlson,S.M., Fuentes,M.E., Francis,J.W., Pillai,R., Lofgren,S.M., Hulea,L., Tandoc,K., Lu,J., Li,A. *et al.* (2019) METTL13 Methylation of eEF1A Increases Translational Output to Promote Tumorigenesis. *Cell*, **176**, 491-504.
23. Cloutier,P., Lavallee-Adam,M., Faubert,D., Blanchette,M. and Coulombe,B. (2013) A newly uncovered group of distantly related lysine methyltransferases preferentially interact with molecular chaperones to regulate their activity. *PLoS.Genet.*, **9**, e1003210.
24. Jakobsson,M.E., Moen,A., Bousset,L., Egge-Jacobsen,W., Kernstock,S., Melki,R. and Falnes,P.O. (2013) Identification and characterization of a novel human methyltransferase modulating Hsp70 function through lysine methylation. *J.Biol.Chem.*, **288**, 27752-27763.
25. Zoabi,M., Zhang,L., Li,T.M., Elias,J.E., Carlson,S.M. and Gozani,O. (2020) Methyltransferase-like 21C (METTL21C) methylates alanine tRNA synthetase at Lys-943 in muscle tissue. *J.Biol.Chem.*, **295**, 11822-11832.
26. Kernstock,S., Davydova,E., Jakobsson,M., Moen,A., Pettersen,S., Maelandsmo,G.M., Egge-Jacobsen,W. and Falnes,P.O. (2012) Lysine methylation of VCP by a member of a novel human protein methyltransferase family. *Nat.Commun.*, **3**, 1038.
27. Chen,D., Ma,H., Hong,H., Koh,S.S., Huang,S.M., Schurter,B.T., Aswad,D.W. and Stallcup,M.R. (1999) Regulation of transcription by a protein methyltransferase. *Science*, **284**, 2174-2177.
28. Hatanaka,Y., Tsusaka,T., Shimizu,N., Morita,K., Suzuki,T., Machida,S., Satoh,M., Honda,A., Hirose,M., Kamimura,S., Ogonuki,N. *et al.* (2017) Histone H3 Methylated at Arginine 17 Is Essential for Reprogramming the Paternal Genome in Zygotes. *Cell Rep.*, **20**, 2756-2765.
29. Rhein,V.F., Carroll,J., Ding,S., Fearnley,I.M. and Walker,J.E. (2013) NDUFAF7 methylates arginine 85 in the NDUFS2 subunit of human complex I. *J Biol.Chem.*, **288**, 33016-33026.
30. Lin,W.J., Gary,J.D., Yang,M.C., Clarke,S. and Herschman,H.R. (1996) The mammalian immediate-early TIS21 protein and the leukemia-associated BTG1 protein interact with a protein-arginine N-methyltransferase. *J Biol.Chem.*, **271**, 15034-15044.
31. Lakowski,T.M. and Frankel,A. (2009) Kinetic analysis of human protein arginine N-methyltransferase 2: formation of monomethyl- and asymmetric dimethyl-arginine residues on histone H4. *Biochem.J*, **421**, 253-261.
32. Swiercz,R., Person,M.D. and Bedford,M.T. (2005) Ribosomal protein S2 is a substrate for mammalian PRMT3 (protein arginine methyltransferase 3). *Biochem.J*, **386**, 85-91.
33. Tang,J., Gary,J.D., Clarke,S. and Herschman,H.R. (1998) PRMT 3, a type I protein arginine N-methyltransferase that differs from PRMT1 in its oligomerization, subcellular localization, substrate specificity, and regulation. *J Biol.Chem.*, **273**, 16935-16945.
34. Branscombe,T.L., Frankel,A., Lee,J.H., Cook,J.R., Yang,Z., Pestka,S. and Clarke,S. (2001) PRMT5 (Janus kinase-binding protein 1) catalyzes the formation of symmetric dimethylarginine residues in proteins. *J Biol.Chem.*, **276**, 32971-32976.
35. Frankel,A., Yadav,N., Lee,J., Branscombe,T.L., Clarke,S. and Bedford,M.T. (2002) The novel human protein arginine N-methyltransferase PRMT6 is a nuclear enzyme displaying unique substrate specificity. *J Biol.Chem.*, **277**, 3537-3543.
36. Miranda,T.B., Miranda,M., Frankel,A. and Clarke,S. (2004) PRMT7 is a member of the protein arginine methyltransferase family with a distinct substrate specificity. *J Biol.Chem.*, **279**, 22902-22907.
37. Bondoc,T.J., Lowe,T.L. and Clarke,S.G. (2023) The exquisite specificity of human protein arginine methyltransferase 7 (PRMT7) toward Arg-X-Arg sites. *PLoS.ONE.*, **18**, e0285812.

38. Lee,J., Sayegh,J., Daniel,J., Clarke,S. and Bedford,M.T. (2005) PRMT8, a new membrane-bound tissue-specific member of the protein arginine methyltransferase family. *J Biol.Chem.*, **280**, 32890-32896.
39. Yang,Y., Hadjikyriacou,A., Xia,Z., Gayatri,S., Kim,D., Zurita-Lopez,C., Kelly,R., Guo,A., Li,W., Clarke,S.G. and Bedford,M.T. (2015) PRMT9 is a type II methyltransferase that methylates the splicing factor SAP145. *Nat.Commun.*, **6**, 6428.
40. De,B., I, Derua,R., Janssens,V., Van,H.C., Waelkens,E., Merlevede,W. and Goris,J. (1999) Purification of porcine brain protein phosphatase 2A leucine carboxyl methyltransferase and cloning of the human homologue. *Biochemistry*, **38**, 16539-16547.
41. Webb,K.J., Lipson,R.S., Al-Hadid,Q., Whitelegge,J.P. and Clarke,S.G. (2010) Identification of protein N-terminal methyltransferases in yeast and humans. *Biochemistry*, **49**, 5225-5235.
42. Tooley,C.E., Petkowski,J.J., Muratore-Schroeder,T.L., Balsbaugh,J.L., Shabanowitz,J., Sabat,M., Minor,W., Hunt,D.F. and Macara,I.G. (2010) NRMT is an alpha-N-methyltransferase that methylates RCC1 and retinoblastoma protein. *Nature*, **466**, 1125-1128.
43. Petkowski,J.J., Bonsignore,L.A., Tooley,J.G., Wilkey,D.W., Merchant,M.L., Macara,I.G. and Schaner Tooley,C.E. (2013) NRMT2 is an N-terminal monomethylase that primes for its homologue NRMT1. *Biochem.J*, **456**, 453-462.
44. Figaro,S., Scrima,N., Buckingham,R.H. and Heurgue-Hamard,V. (2008) HemK2 protein, encoded on human chromosome 21, methylates translation termination factor eRF1. *FEBS Lett.*, **582**, 2352-2356.
45. Ishizawa,T., Nozaki,Y., Ueda,T. and Takeuchi,N. (2008) The human mitochondrial translation release factor HMRF1L is methylated in the GGQ motif by the methyltransferase HMPrmC. *Biochem.Biophys.Res.Commun.*, **373**, 99-103.

Supplementary Table 2. Human DNA/RNA (seven-β-strand) MTases

For a given MTase, the listed reference(s) describes the characterization of the MTase in the indicated year (which is also used in the timeline in Fig. 2A). The information on substrates/products are based on these references, as well as on original articles and reviews referred to in the main paper. The MTases have been grouped according to their products, with m^6A-, m^5C- and Xm-generating MTases as the largest groups, and these have been shaded in green, blue and red, respectively. Within each subgroup the MTases are alphabetically sorted on gene symbol.

UniProt	Gene symbol	Alias	Status	UniProt name	Substrate(s)	(Likely) product	Year	Refs.
O43159	RRP8	NML	Est	Ribosomal RNA-processing protein 8	A1322 in 28S rRNA	m^1A	2016	(1,2)
Q96FX7	TRMT61A	TRM61	Est	tRNA (adenine(58)-N(1))-methyltransferase catalytic subunit TRMT61A	A58 in cytosolic tRNAs	m^1A	2005	(3)
Q9BVS5	TRMT61B		Est	tRNA (adenine(58)-N(1))-methyltransferase, mitochondrial	A58 in mt-tRNA and A947 in 16S mt-rRNA	m^1A	2012	(4,5)
Q86U44	METTL3	MTA70	Est	N(6)-adenosine-methyltransferase catalytic subunit	mRNA	m^6A	2014	(6)
Q8N3J2	METTL4		Est	N(6)-adenine-specific methyltransferase METTL4	Am in pos. 30 of U2 snRNA.	m^6Am	2020	(7,8)
Q9NRN9	METTL5		Est	rRNA N(6)-adenosine-methyltransferase METTL5	A1832 in 18S rRNA	m^6A	2019	(9)
Q9HCE5	METTL14	KIAA1627	Est	N(6)-adenosine-methyltransferase non-catalytic subunit	mRNA	m^6A	2014	(6)
Q86W50	METTL16	METT10D	Est	RNA N(6)-adenosine-methyltransferase METTL16	mRNA, A43 in U6 snRNA	m^6A	2017	(10)
Q9H4Z3	PCIF1	CAPAM	Est	mRNA (2'-O-methyladenosine-N(6)-)-methyltransferase	Am at mRNA cap	m^6Am	2018	(11-14)
Q9H5U6	ZCCHC4		Est	rRNA N(6)-adenosine-methyltransferase ZCCHC4	A4220 in 28S rRNA	m^6A	2018	(9,15,16)
Q9UNQ2	DIMT1	DIMT1L	Est	Dimethyladenosine transferase	A1850 and A1851 in 18S rRNA	m^6_2A	2015	(17)
Q8WVM0	TFB1M	MTTFB	Est	Dimethyladenosine transferase 1, mitochondrial	A1006 and A1007 in 12S mt-rRNA	m^6_2A	2009	(18)
Q9H5Q4	TFB2M		Tent	Dimethyladenosine transferase 2, mitochondrial	?	m^6_2A		
Q96IZ6	METTL2A	METTL2	Est	tRNA N(3)-methylcytidine methyltransferase METTL2A	C32 in tRNA-Thr/Arg	m^3C	2017	(19,20)
Q6P1Q9	METTL2B		Est	tRNA N(3)-methylcytidine methyltransferase METTL2B	Similar to METTL2A, low activity	m^3C	2017	(19,20)
Q8TCB7	METTL6		Est	tRNA N(3)-methylcytidine methyltransferase METTL6	C32 in tRNA-Ser	m^3C	2017	(19-21)
Q9H825	METTL8		Est	tRNA N(3)-methylcytidine methyltransferase METTL8, mitochondrial	C32 in mt-tRNA-Thr/Ser(UCN)	m^3C	2017	(20,22,23)
A6NJ78	METTL15		Est	12S rRNA N(4)-methylcytidine (m4C) methyltransferase	C839 in 12S mt-rRNA	m^4C	2019	(24)
P26358	DNMT1		Est	DNA (cytosine-5)-methyltransferase 1	CG in DNA (maintenance MTase)	m^5C	1988	(25)
Q9Y6K1	DNMT3A		Est	DNA (cytosine-5)-methyltransferase 3A	CG in DNA (*de novo* MTase)	m^5C	1998	(26)
Q9UBC3	DNMT3B		Est	DNA (cytosine-5)-methyltransferase 3B	CG in DNA (*de novo* MTase)	m^5C	1998	(26)
Q9UJW3	DNMT3L		Est	DNA (cytosine-5)-methyltransferase 3-like	CG in DNA (*de novo* MTase)	m^5C	2007	(27)
P46087	NOP2	NOL1, NSUN1	Est	28S rRNA (cytosine(4447)-C(5))-methyltransferase	C4447 in 28S rRNA	m^5C	2015	(28)
Q08J23	NSUN2	TRM4	Est	RNA cytosine C(5)-methyltransferase NSUN2	C34 in pre-tRNA Leu (CAA)	m^5C	2006	(29)
Q9H649	NSUN3		Est	tRNA (cytosine(34)-C(5))-methyltransferase, mitochondrial	C34 in tRNA(Met)	m^5C	2016	(30,31)
Q96CB9	NSUN4		Est	5-methylcytosine rRNA methyltransferase NSUN4	C911 in 12S mt-rRNA	m^5C	2014	(32)
Q96P11	NSUN5	WBSCR20	Est	28S rRNA (cytosine-C(5))-methyltransferase	C3782 in 28S ribosomal RNA	m^5C	2019	(33)
Q8TEA1	NSUN6		Est	tRNA (cytosine(72)-C(5))-methyltransferase NSUN6	C72 in tRNA(Cys) and tRNA(Thr)	m^5C	2015	(34)

Q8NE18	NSUN7		Tent	Putative methyltransferase NSUN7	?	m^5C		
O14717	TRDMT1	DNMT2	Est	tRNA (cytosine(38)-C(5))-methyltransferase	C38 in tRNA-Asp(GUC)/(Gly(GCC)/Val(AAC)	m^5C	2006	(35)
Q32P41	TRMT5	TRM5	Est	tRNA (guanine(37)-N1)-methyltransferase	G37 in many tRNAs	m^1G	2004	(36)
Q9BTF0	THUMPD2	C2orf8	Est	THUMP domain-containing protein 2	G72 in U6 snRNA	m^2G	2023	(37)
Q9BV44	THUMPD3		Est	tRNA (guanine(6)-N2)-methyltransferase THUMP3	G6 in several tRNAs	m^2G	2021	(38)
Q7Z4G4	TRMT11		Est	tRNA (guanine(10)-N2)-methyltransferase homolog	G10 in several tRNAs	m^2G	2023	(37)
Q9NXH9	TRMT1	TRM1	Est	tRNA (guanine(26)-N(2))-dimethyltransferase	G26 in several tRNAs	m^2_2G	2000	(39)
Q7Z2T5	TRMT1L	C1orf25	Tent	TRMT1-like protein	?	m^2_2G		
Q96RS0	TGS1	PIMT	Est	Trimethylguanosine synthase	m^7G at the 5' cap of snRNAs and snoRNAs	$m^{2,2,7}G$	2008	(40)
O43709	BUD23	MERM1, WBSCR22	Est	18S rRNA (guanine-N(7))-methyltransferase	G1639 in 18S rRNA	m^7G	2015	(17)
Q9UBP6	METTL1		Est	tRNA (guanine-N(7)-)-methyltransferase	G46 in several tRNAs	m^7G	2005	(41)
O43148	RNMT		Est	mRNA cap guanine-N7 methyltransferase	G in mRNA cap	m^7G	1998	(42)
O60294	LCMT2	TYW4	Tent	tRNA wybutosine-synthesizing protein 4	yW precursor in pos. 37 in tRNA?	yW		(43)
Q8IZ69	TRMT2A		Est	tRNA (uracil-5-)-methyltransferase homolog A	U54 in several tRNAs	m^5U	2019	(44)
Q96GJ1	TRMT2B		Est	RNA (uracil-5-)-methyltransferase homolog B	U54 in mt-tRNA and U429 in 12S mt-rRNA	m^5U	2020	(45,46)
Q96BT7	ALKBH8	ABH8, TRM9	Est	Alkylated DNA repair protein alkB homolog 8	Wobble uridine (U34) in various tRNAs	mcm^5U	2010	(47,48)
Q9P272	TRMT9B	KIAA1456	Tent	Probable tRNA methyltransferase 9-like protein	Wobble uridine (U34) in tRNA?	mcm^5U		(49)
Q8N1G2	CMTR1	FTSJD2, MTR1	Est	Cap-specific mRNA (nucleoside-2'-O-)-methyltransferase 1	1st mRNA nucleotide (Cap 1)	Xm	2010	(50)
Q8IYT2	CMTR2	FTSJD1	Est	Cap-specific mRNA (nucleoside-2'-O-)-methyltransferase 2	2nd mRNA nucleotide (Cap 2)	Xm	2011	(51)
P22087	FBL		Est	rRNA 2'-O-methyltransferase fibrillarin	rRNA (several sites)	Xm	1991	(52)
A6NHQ2	FBLL1		Tent	rRNA/tRNA 2'-O-methyltransferase fibrillarin-like protein 1	?	Xm		
Q9UET6	FTSJ1	MRX9	Est	tRNA (cytidine(32)/guanosine(34)-2'-O)-methyltransferase	Pos. 32 and 34 in tRNA	Xm	2015	(53,54)
Q8IY81	FTSJ3		Est	pre-rRNA processing protein FTSJ3	HIV RNA	Xm	2019	(55)
Q5T8I9	HENMT1		Est	Small RNA 2'-O-methyltransferase	3'-terminal nucleotide of piRNAs	Xm	2007	(56)
Q9UI43	MRM2	FTSJ2	Est	rRNA methyltransferase 2, mitochondrial	U1369 of 16S mt-rRNA	Xm	2014	(57)
Q9NUP7	TRMT13		Est	tRNA:m(4)X modification enzyme TRM13 homolog	Pos. 4 in tRNA	Xm	2022	(58)
Q8IYL2	TRMT44	METTL19	Tent	Probable tRNA (uracil-O(2)-)-methyltransferase	U44 in tRNA-Ser?	Xm		(59)
Q7Z5W3	BCDIN3D		Est	RNA 5'-monophosphate methyltransferase	5' monophosphate of tRNA-His	α-Pme1	2012	(60,61)
Q7L2J0	MEPCE	BCDIN3	Est	7SK snRNA methylphosphate capping enzyme	5' triphosphate on 7SK snRNA	γ-Pme1	2007	(62)

Abbreviations: mt-rRNA. mitochondrial rRNA, α-Pme1, methyl ester of α-phosphate on the 5'end of RNA; γ-Pme1, methyl ester of γ-phosphate on the 5'end of RNA; Est, established; mcm^5U, methoxycarbonylmethyluridine, Tent, tentative; yW, wybutosine

References for Supplementary Table 2

1. Sharma,S., Hartmann,J.D., Watzinger,P., Klepper,A., Peifer,C., Kotter,P., Lafontaine,D.L.J. and Entian,K.D. (2018) A single N(1)-methyladenosine on the large ribosomal subunit rRNA impacts locally its structure and the translation of key metabolic enzymes. *Sci.Rep.*, **8**, 11904.
2. Waku,T., Nakajima,Y., Yokoyama,W., Nomura,N., Kako,K., Kobayashi,A., Shimizu,T. and Fukamizu,A. (2016) NML-mediated rRNA base methylation links ribosomal subunit formation to cell proliferation in a p53-dependent manner. *J Cell Sci.*, **129**, 2382-2393.

3. Ozanick,S., Krecic,A., Andersland,J. and Anderson,J.T. (2005) The bipartite structure of the tRNA m1A58 methyltransferase from S. cerevisiae is conserved in humans. *RNA.*, **11**, 1281-1290.
4. Bar-Yaacov,D., Frumkin,I., Yashiro,Y., Chujo,T., Ishigami,Y., Chemla,Y., Blumberg,A., Schlesinger,O., Bieri,P., Greber,B., Ban,N. *et al.* (2016) Mitochondrial 16S rRNA Is Methylated by tRNA Methyltransferase TRMT61B in All Vertebrates. *PLoS.Biol.*, **14**, e1002557.
5. Chujo,T. and Suzuki,T. (2012) Trmt61B is a methyltransferase responsible for 1-methyladenosine at position 58 of human mitochondrial tRNAs. *RNA.*, **18**, 2269-2276.
6. Liu,J., Yue,Y., Han,D., Wang,X., Fu,Y., Zhang,L., Jia,G., Yu,M., Lu,Z., Deng,X., Dai,Q. *et al.* (2014) A METTL3-METTL14 complex mediates mammalian nuclear RNA N6-adenosine methylation. *Nat.Chem.Biol.*, **10**, 93-95.
7. Chen,H., Gu,L., Orellana,E.A., Wang,Y., Guo,J., Liu,Q., Wang,L., Shen,Z., Wu,H., Gregory,R.I., Xing,Y. *et al.* (2020) METTL4 is an snRNA m(6)Am methyltransferase that regulates RNA splicing. *Cell Res.*, **30**, 544-547.
8. Goh,Y.T., Koh,C.W.Q., Sim,D.Y., Roca,X. and Goh,W.S.S. (2020) METTL4 catalyzes m6Am methylation in U2 snRNA to regulate pre-mRNA splicing. *Nucleic Acids Res.*, **48**, 9250-9261.
9. van Tran,N., Ernst,F.G.M., Hawley,B.R., Zorbas,C., Ulryck,N., Hackert,P., Bohnsack,K.E., Bohnsack,M.T., Jaffrey,S.R., Graille,M. and Lafontaine,D.L.J. (2019) The human 18S rRNA m6A methyltransferase METTL5 is stabilized by TRMT112. *Nucleic Acids Res.*, **47**, 7719-7733.
10. Pendleton,K.E., Chen,B., Liu,K., Hunter,O.V., Xie,Y., Tu,B.P. and Conrad,N.K. (2017) The U6 snRNA m(6)A Methyltransferase METTL16 Regulates SAM Synthetase Intron Retention. *Cell*, **169**, 824-835.
11. Sendinc,E., Valle-Garcia,D., Dhall,A., Chen,H., Henriques,T., Navarrete-Perea,J., Sheng,W., Gygi,S.P., Adelman,K. and Shi,Y. (2019) PCIF1 Catalyzes m6Am mRNA Methylation to Regulate Gene Expression. *Mol.Cell*, **75**, 620-630.
12. Boulias,K., Toczydlowska-Socha,D., Hawley,B.R., Liberman,N., Takashima,K., Zaccara,S., Guez,T., Vasseur,J.J., Debart,F., Aravind,L., Jaffrey,S.R. *et al.* (2019) Identification of the m(6)Am Methyltransferase PCIF1 Reveals the Location and Functions of m(6)Am in the Transcriptome. *Mol.Cell*, **75**, 631-643.
13. Akichika,S., Hirano,S., Shichino,Y., Suzuki,T., Nishimasu,H., Ishitani,R., Sugita,A., Hirose,Y., Iwasaki,S., Nureki,O. and Suzuki,T. (2018) Cap-specific terminal N (6)-methylation of RNA by an RNA polymerase II-associated methyltransferase. *Science*, **363**, eaav0080.
14. Sun,H., Zhang,M., Li,K., Bai,D. and Yi,C. (2018) Cap-specific, terminal N(6)-methylation by a mammalian m(6)Am methyltransferase. *Cell Res.*, **29**, 80-82.
15. Ma,H., Wang,X., Cai,J., Dai,Q., Natchiar,S.K., Lv,R., Chen,K., Lu,Z., Chen,H., Shi,Y.G., Lan,F. *et al.* (2019) N(6-)Methyladenosine methyltransferase ZCCHC4 mediates ribosomal RNA methylation. *Nat.Chem.Biol.*, **15**, 88-94.
16. Pinto,R., Vagbo,C.B., Jakobsson,M.E., Kim,Y., Baltissen,M.P., O'Donohue,M.F., Guzman,U.H., Malecki,J.M., Wu,J., Kirpekar,F., Olsen,J.V. *et al.* (2020) The human methyltransferase ZCCHC4 catalyses N6-methyladenosine modification of 28S ribosomal RNA. *Nucleic Acids Res.*, **48**, 830-846.
17. Zorbas,C., Nicolas,E., Wacheul,L., Huvelle,E., Heurgue-Hamard,V. and Lafontaine,D.L. (2015) The human 18S rRNA base methyltransferases DIMT1L and WBSCR22-TRMT112 but not rRNA modification are required for ribosome biogenesis. *Mol.Biol.Cell*, **26**, 2080-2095.
18. Metodiev,M.D., Lesko,N., Park,C.B., Camara,Y., Shi,Y., Wibom,R., Hultenby,K., Gustafsson,C.M. and Larsson,N.G. (2009) Methylation of 12S rRNA is necessary for in vivo stability of the small subunit of the mammalian mitochondrial ribosome. *Cell Metab*, **9**, 386-397.
19. Mao,X.L., Li,Z.H., Huang,M.H., Wang,J.T., Zhou,J.B., Li,Q.R., Xu,H., Wang,X.J. and Zhou,X.L. (2021) Mutually exclusive substrate selection strategy by human m3C RNA transferases METTL2A and METTL6. *Nucleic Acids Res.*, **49**, 8309-8323.
20. Xu,L., Liu,X., Sheng,N., Oo,K.S., Liang,J., Chionh,Y.H., Xu,J., Ye,F., Gao,Y.G., Dedon,P.C. and Fu,X.Y. (2017) Three distinct 3-methylcytidine (m(3)C) methyltransferases modify tRNA and mRNA in mice and humans. *J Biol.Chem.*, **292**, 14695-14703.
21. Ignatova,V.V., Kaiser,S., Ho,J.S.Y., Bing,X., Stolz,P., Tan,Y.X., Lee,C.L., Gay,F.P.H., Lastres,P.R., Gerlini,R., Rathkolb,B. *et al.* (2020) METTL6 is a tRNA m(3)C methyltransferase that regulates pluripotency and tumor cell growth. *Sci.Adv.*, **6**, eaaz4551.
22. Kleiber,N., Lemus-Diaz,N., Stiller,C., Heinrichs,M., Mai,M.M., Hackert,P., Richter-Dennerlein,R., Hobartner,C., Bohnsack,K.E. and Bohnsack,M.T. (2022) The RNA methyltransferase METTL8 installs m(3)C(32) in mitochondrial tRNAs(Thr/Ser(UCN)) to optimise tRNA structure and mitochondrial translation. *Nat.Commun.*, **13**, 209.
23. Scholler,E., Marks,J., Marchand,V., Bruckmann,A., Powell,C.A., Reichold,M., Mutti,C.D., Dettmer,K., Feederle,R., Huttelmaier,S., Helm,M. *et al.* (2021) Balancing of mitochondrial translation through METTL8-mediated m(3)C modification of mitochondrial tRNAs. *Mol.Cell*, **81**, 4810-4825.

24. Van,H.L., Hendrick,A.G., D'Souza,A.R., Powell,C.A., Rebelo-Guiomar,P., Harbour,M.E., Ding,S., Fearnley,I.M., Andrews,B. and Minczuk,M. (2019) METTL15 introduces N4-methylcytidine into human mitochondrial 12S rRNA and is required for mitoribosome biogenesis. *Nucleic Acids Res.*, **47**, 10267-10281.
25. Bestor,T., Laudano,A., Mattaliano,R. and Ingram,V. (1988) Cloning and sequencing of a cDNA encoding DNA methyltransferase of mouse cells. The carboxyl-terminal domain of the mammalian enzymes is related to bacterial restriction methyltransferases. *J Mol.Biol.*, **203**, 971-983.
26. Okano,M., Xie,S. and Li,E. (1998) Cloning and characterization of a family of novel mammalian DNA (cytosine-5) methyltransferases. *Nat.Genet.*, **19**, 219-220.
27. Ooi,S.K., Qiu,C., Bernstein,E., Li,K., Jia,D., Yang,Z., Erdjument-Bromage,H., Tempst,P., Lin,S.P., Allis,C.D., Cheng,X. *et al.* (2007) DNMT3L connects unmethylated lysine 4 of histone H3 to de novo methylation of DNA. *Nature*, **448**, 714-717.
28. Bourgeois,G., Ney,M., Gaspar,I., Aigueperse,C., Schaefer,M., Kellner,S., Helm,M. and Motorin,Y. (2015) Eukaryotic rRNA Modification by Yeast 5-Methylcytosine-Methyltransferases and Human Proliferation-Associated Antigen p120. *PLoS.ONE.*, **10**, e0133321.
29. Brzezicha,B., Schmidt,M., Makalowska,I., Jarmolowski,A., Pienkowska,J. and Szweykowska-Kulinska,Z. (2006) Identification of human tRNA:m5C methyltransferase catalysing intron-dependent m5C formation in the first position of the anticodon of the pre-tRNA Leu (CAA). *Nucleic Acids Res.*, **34**, 6034-6043.
30. Haag,S., Sloan,K.E., Ranjan,N., Warda,A.S., Kretschmer,J., Blessing,C., Hubner,B., Seikowski,J., Dennerlein,S., Rehling,P., Rodnina,M.V. *et al.* (2016) NSUN3 and ABH1 modify the wobble position of mt-tRNAMet to expand codon recognition in mitochondrial translation. *EMBO J.*, **35**, 2104-2119.
31. Nakano,S., Suzuki,T., Kawarada,L., Iwata,H., Asano,K. and Suzuki,T. (2016) NSUN3 methylase initiates 5-formylcytidine biogenesis in human mitochondrial tRNA(Met). *Nat.Chem.Biol.*, **12**, 546-551.
32. Metodiev,M.D., Spahr,H., Loguercio,P.P., Meharg,C., Becker,C., Altmueller,J., Habermann,B., Larsson,N.G. and Ruzzenente,B. (2014) NSUN4 is a dual function mitochondrial protein required for both methylation of 12S rRNA and coordination of mitoribosomal assembly. *PLoS.Genet.*, **10**, e1004110.
33. Heissenberger,C., Liendl,L., Nagelreiter,F., Gonskikh,Y., Yang,G., Stelzer,E.M., Krammer,T.L., Micutkova,L., Vogt,S., Kreil,D.P., Sekot,G. *et al.* (2019) Loss of the ribosomal RNA methyltransferase NSUN5 impairs global protein synthesis and normal growth. *Nucleic Acids Res.*, **47**, 11807-11825.
34. Haag,S., Warda,A.S., Kretschmer,J., Gunnigmann,M.A., Hobartner,C. and Bohnsack,M.T. (2015) NSUN6 is a human RNA methyltransferase that catalyzes formation of m5C72 in specific tRNAs. *RNA.*, **21**, 1532-1543.
35. Goll,M.G., Kirpekar,F., Maggert,K.A., Yoder,J.A., Hsieh,C.L., Zhang,X., Golic,K.G., Jacobsen,S.E. and Bestor,T.H. (2006) Methylation of tRNAAsp by the DNA methyltransferase homolog Dnmt2. *Science*, **311**, 395-398.
36. Brule,H., Elliott,M., Redlak,M., Zehner,Z.E. and Holmes,W.M. (2004) Isolation and characterization of the human tRNA-(N1G37) methyltransferase (TRM5) and comparison to the Escherichia coli TrmD protein. *Biochemistry*, **43**, 9243-9255.
37. Wang,C., Ulryck,N., Herzel,L., Pythoud,N., Kleiber,N., Guerineau,V., Jactel,V., Moritz,C., Bohnsack,M.T., Carapito,C., Touboul,D. *et al.* (2023) N 2-methylguanosine modifications on human tRNAs and snRNA U6 are important for cell proliferation, protein translation and pre-mRNA splicing. *Nucleic Acids Res.*, **51**, 7496-7519.
38. Yang,W.Q., Xiong,Q.P., Ge,J.Y., Li,H., Zhu,W.Y., Nie,Y., Lin,X., Lv,D., Li,J., Lin,H. and Liu,R.J. (2021) THUMPD3-TRMT112 is a m2G methyltransferase working on a broad range of tRNA substrates. *Nucleic Acids Res.*, **49**, 11900-11919.
39. Liu,J. and Straby,K.B. (2000) The human tRNA(m(2)(2)G(26))dimethyltransferase: functional expression and characterization of a cloned hTRM1 gene. *Nucleic Acids Res.*, **28**, 3445-3451.
40. Girard,C., Verheggen,C., Neel,H., Cammas,A., Vagner,S., Soret,J., Bertrand,E. and Bordonne,R. (2008) Characterization of a short isoform of human Tgs1 hypermethylase associating with small nucleolar ribonucleoprotein core proteins and produced by limited proteolytic processing. *J Biol.Chem.*, **283**, 2060-2069.
41. Cartlidge,R.A., Knebel,A., Peggie,M., Alexandrov,A., Phizicky,E.M. and Cohen,P. (2005) The tRNA methylase METTL1 is phosphorylated and inactivated by PKB and RSK in vitro and in cells. *EMBO J.*, **24**, 1696-1705.
42. Tsukamoto,T., Shibagaki,Y., Niikura,Y. and Mizumoto,K. (1998) Cloning and characterization of three human cDNAs encoding mRNA (guanine-7-)-methyltransferase, an mRNA cap methylase. *Biochem.Biophys.Res.Commun.*, **251**, 27-34.
43. Noma,A., Kirino,Y., Ikeuchi,Y. and Suzuki,T. (2006) Biosynthesis of wybutosine, a hyper-modified nucleoside in eukaryotic phenylalanine tRNA. *EMBO J.*, **25**, 2142-2154.
44. Carter,J.M., Emmett,W., Mozos,I.R., Kotter,A., Helm,M., Ule,J. and Hussain,S. (2019) FICC-Seq: a method for enzyme-specified profiling of methyl-5-uridine in cellular RNA. *Nucleic Acids Res.*, **47**, e113.

45. Laptev,I., Shvetsova,E., Levitskii,S., Serebryakova,M., Rubtsova,M., Bogdanov,A., Kamenski,P., Sergiev,P. and Dontsova,O. (2020) Mouse Trmt2B protein is a dual specific mitochondrial metyltransferase responsible for m(5)U formation in both tRNA and rRNA. *RNA.Biol.*, **17**, 441-450.
46. Powell,C.A. and Minczuk,M. (2020) TRMT2B is responsible for both tRNA and rRNA m(5)U-methylation in human mitochondria. *RNA.Biol.*, **17**, 451-462.
47. Fu,D., Brophy,J.A., Chan,C.T., Atmore,K.A., Begley,U., Paules,R.S., Dedon,P.C., Begley,T.J. and Samson,L.D. (2010) Human AlkB homolog ABH8 Is a tRNA methyltransferase required for wobble uridine modification and DNA damage survival. *Mol.Cell Biol.*, **30**, 2449-2459.
48. Songe-Moller,L., van den Born,E., Leihne,V., Vagbo,C.B., Kristoffersen,T., Krokan,H.E., Kirpekar,F., Falnes,P.O. and Klungland,A. (2010) Mammalian ALKBH8 possesses tRNA methyltransferase activity required for the biogenesis of multiple wobble uridine modifications implicated in translational decoding. *Mol.Cell Biol.*, **30**, 1814-1827.
49. Begley,U., Sosa,M.S., Avivar-Valderas,A., Patil,A., Endres,L., Estrada,Y., Chan,C.T., Su,D., Dedon,P.C., Aguirre-Ghiso,J.A. and Begley,T. (2013) A human tRNA methyltransferase 9-like protein prevents tumour growth by regulating LIN9 and HIF1-alpha. *EMBO Mol.Med.*, **5**, 366-383.
50. Belanger,F., Stepinski,J., Darzynkiewicz,E. and Pelletier,J. (2010) Characterization of hMTr1, a human Cap1 2'-O-ribose methyltransferase. *J Biol.Chem.*, **285**, 33037-33044.
51. Werner,M., Purta,E., Kaminska,K.H., Cymerman,I.A., Campbell,D.A., Mittra,B., Zamudio,J.R., Sturm,N.R., Jaworski,J. and Bujnicki,J.M. (2011) 2'-O-ribose methylation of cap2 in human: function and evolution in a horizontally mobile family. *Nucleic Acids Res.*, **39**, 4756-4768.
52. Jansen,R.P., Hurt,E.C., Kern,H., Lehtonen,H., Carmo-Fonseca,M., Lapeyre,B. and Tollervey,D. (1991) Evolutionary conservation of the human nucleolar protein fibrillarin and its functional expression in yeast. *J Cell Biol.*, **113**, 715-729.
53. Guy,M.P., Shaw,M., Weiner,C.L., Hobson,L., Stark,Z., Rose,K., Kalscheuer,V.M., Gecz,J. and Phizicky,E.M. (2015) Defects in tRNA Anticodon Loop 2'-O-Methylation Are Implicated in Nonsyndromic X-Linked Intellectual Disability due to Mutations in FTSJ1. *Hum.Mutat.*, **36**, 1176-1187.
54. Li,J., Wang,Y.N., Xu,B.S., Liu,Y.P., Zhou,M., Long,T., Li,H., Dong,H., Nie,Y., Chen,P.R., Wang,E.D. *et al.* (2020) Intellectual disability-associated gene ftsj1 is responsible for 2'-O-methylation of specific tRNAs. *EMBO Rep.*, **21**, e50095.
55. Ringeard,M., Marchand,V., Decroly,E., Motorin,Y. and Bennasser,Y. (2019) FTSJ3 is an RNA 2'-O-methyltransferase recruited by HIV to avoid innate immune sensing. *Nature*, **565**, 500-504.
56. Kirino,Y. and Mourelatos,Z. (2007) The mouse homolog of HEN1 is a potential methylase for Piwi-interacting RNAs. *RNA.*, **13**, 1397-1401.
57. Lee,K.W. and Bogenhagen,D.F. (2014) Assignment of 2'-O-methyltransferases to modification sites on the mammalian mitochondrial large subunit 16 S ribosomal RNA (rRNA). *J Biol.Chem.*, **289**, 24936-24942.
58. Li,H., Dong,H., Xu,B., Xiong,Q.P., Li,C.T., Yang,W.Q., Li,J., Huang,Z.X., Zeng,Q.Y., Wang,E.D. and Liu,R.J. (2022) A dual role of human tRNA methyltransferase hTrmt13 in regulating translation and transcription. *EMBO J.*, **41**, e108544.
59. Kotelawala,L., Grayhack,E.J. and Phizicky,E.M. (2008) Identification of yeast tRNA Um(44) 2'-O-methyltransferase (Trm44) and demonstration of a Trm44 role in sustaining levels of specific tRNA(Ser) species. *RNA.*, **14**, 158-169.
60. Martinez,A., Yamashita,S., Nagaike,T., Sakaguchi,Y., Suzuki,T. and Tomita,K. (2017) Human BCDIN3D monomethylates cytoplasmic histidine transfer RNA. *Nucleic Acids Res.*, **45**, 5423-5436.
61. Xhemalce,B., Robson,S.C. and Kouzarides,T. (2012) Human RNA methyltransferase BCDIN3D regulates microRNA processing. *Cell*, **151**, 278-288.
62. Jeronimo,C., Forget,D., Bouchard,A., Li,Q., Chua,G., Poitras,C., Therien,C., Bergeron,D., Bourassa,S., Greenblatt,J., Chabot,B. *et al.* (2007) Systematic analysis of the protein interaction network for the human transcription machinery reveals the identity of the 7SK capping enzyme. *Mol.Cell*, **27**, 262-274.

Supplementary Table 3. Human small molecule (seven-β-strand) MTases

For a given MTase, the listed reference(s) correspond to the characterisation of the MTase in the indicated year (which is also the basis for Fig. 2A). The information on substrates/products are based on these references, as well as on original articles and reviews referred to in the main paper. Color shading indicate MTase subgroups, i.e. neurotransmitter (yellow), metabolite (green), detoxifying (blue) or putative (grey) MTases.

UniProt ID	Gene symbol	Alias	Status	UniProt name	Prototype substrate	Product	Year	Ref.
P46597	ASMT	HIOMT	Est	Acetylserotonin O-methyltransferase	N-acetylserotonin	Melatonin (N-acetyl-5-methoxy tryptamine)	1992	(1)
P21964	COMT		Est	Catechol O-methyltransferase	Dopamine	3-methoxytyramine	1991	(2)
P50135	HNMT		Est	Histamine N-methyltransferase	Histamine	N(tau)-methylhistamine	1994	(3)
O95050	INMT		Est	Indolethylamine N-methyltransferase	Tryptamine	N-methyltryptamine	1999	(4)
P11086	PNMT		Est	Phenylethanolamine N-methyltransferase	Norepinephrine	Epinephrine	1988	(5)
Q9NZJ6	COQ3		Est	Ubiquinone biosynthesis O-methyltransferase, mitochondrial	DMeQ	CoQ	2000	(6)
Q5HYK3	COQ5		Est	2-methoxy-6-polyprenyl-1,4-benzoquinol methylase, mitochondrial	DDMQ	DMQ	2014	(7)
Q14353	GAMT		Est	Guanidinoacetate N-methyltransferase	Guanidinoacetate	Creatine	1988	(8)
Q14749	GNMT		Est	Glycine N-methyltransferase	Glycine	Sarcosine	1997	(9)
P40261	NNMT		Est	Nicotinamide N-methyltransferase	Nicotinamide	1-methylnicotinamide	1994	(10)
P51580	TPMT		Est	Thiopurine S-methyltransferase	6-mercaptopurine<br>Molybdopterin	6-methylmercaptopurine<br>Urothione	1993<br>2023	(11)<br>(12)
Q9HBK9	AS3MT		Est	Arsenite methyltransferase	Arsenate (As[V])	Dimethylarsinate	2002	(13)
Q8N4J0	CARNMT1		Est	Carnosine N-methyltransferase	Carnosine	Anserine	2015	(14)
Q9H8H3	TMT1A	METTL7A	Est	Thiol S-methyltransferase TMT1A	Alkyl thiol	Alkyl methyl thioether	2023	(15)
Q6UX53	TMT1B	METTL7B	Est	Thiol S-methyltransferase TMT1B	Alkyl thiol	Alkyl methyl thioether	2021	(16)
O95671	ASMTL		Tent	Probable bifunctional dTTP/UTP pyrophosphatase/methyltransferase protein				
Q86VU5	COMTD1		Tent	Catechol O-methyltransferase domain-containing protein 1				

Abbreviations: DDMQ, demethoxy-demethyl-coenzyme Q; DMQ, demethoxy-coenzyme Q; DMeQ, demethyl-coenzyme Q; CoQ, coenzyme Q; Est, established; Tent, tentative

References for Supplementary Table 3

1. Donohue,S.J., Roseboom,P.H. and Klein,D.C. (1992) Bovine hydroxyindole-O-methyltransferase. Significant sequence revision. *J Biol.Chem.*, **267**, 5184-5185.
2. Bertocci,B., Miggiano,V., Da,P.M., Dembic,Z., Lahm,H.W. and Malherbe,P. (1991) Human catechol-O-methyltransferase: cloning and expression of the membrane-associated form. *Proc.Natl.Acad.Sci.U.S.A*, **88**, 1416-1420.
3. Girard,B., Otterness,D.M., Wood,T.C., Honchel,R., Wieben,E.D. and Weinshilboum,R.M. (1994) Human histamine N-methyltransferase pharmacogenetics: cloning and expression of kidney cDNA. *Mol.Pharmacol.*, **45**, 461-468.
4. Thompson,M.A., Moon,E., Kim,U.J., Xu,J., Siciliano,M.J. and Weinshilboum,R.M. (1999) Human indolethylamine N-methyltransferase: cDNA cloning and expression, gene cloning, and chromosomal localization. *Genomics*, **61**, 285-297.
5. Kaneda,N., Ichinose,H., Kobayashi,K., Oka,K., Kishi,F., Nakazawa,A., Kurosawa,Y., Fujita,K. and Nagatsu,T. (1988) Molecular cloning of cDNA and chromosomal assignment of the gene for human phenylethanolamine N-methyltransferase, the enzyme for epinephrine biosynthesis. *J Biol.Chem.*, **263**, 7672-7677.
6. Jonassen,T. and Clarke,C.F. (2000) Isolation and functional expression of human COQ3, a gene encoding a methyltransferase required for ubiquinone biosynthesis. *J Biol.Chem.*, **275**, 12381-12387.
7. Nguyen,T.P., Casarin,A., Desbats,M.A., Doimo,M., Trevisson,E., Santos-Ocana,C., Navas,P., Clarke,C.F. and Salviati,L. (2014) Molecular characterization of the human COQ5 C-methyltransferase in coenzyme Q10 biosynthesis. *Biochim.Biophys.Acta*, **1841**, 1628-1638.
8. Ogawa,H., Date,T., Gomi,T., Konishi,K., Pitot,H.C., CANTONI,G.L. and Fujioka,M. (1988) Molecular cloning, sequence analysis, and expression in Escherichia coli of the cDNA for guanidinoacetate methyltransferase from rat liver. *Proc.Natl.Acad.Sci.U.S.A*, **85**, 694-698.
9. Ogawa,H., Gomi,T., Takata,Y., Date,T. and Fujioka,M. (1997) Recombinant expression of rat glycine N-methyltransferase and evidence for contribution of N-terminal acetylation to co-operative binding of S-adenosylmethionine. *Biochem.J*, **327 (Pt 2)**, 407-412.
10. Aksoy,S., Szumlanski,C.L. and Weinshilboum,R.M. (1994) Human liver nicotinamide N-methyltransferase. cDNA cloning, expression, and biochemical characterization. *J Biol.Chem.*, **269**, 14835-14840.
11. Honchel,R., Aksoy,I.A., Szumlanski,C., Wood,T.C., Otterness,D.M., Wieben,E.D. and Weinshilboum,R.M. (1993) Human thiopurine methyltransferase: molecular cloning and expression of T84 colon carcinoma cell cDNA. *Mol.Pharmacol.*, **43**, 878-887.
12. Pristup,J., Schaeffeler,E., Arjune,S., Hofmann,U., Angel Santamaria-Araujo,J., Leuthold,P., Friedrich,N., Nauck,M., Mayr,S., Haag,M., Muerdter,T. *et al.* (2022) Molybdenum Cofactor Catabolism Unravels the Physiological Role of the Drug Metabolizing Enzyme Thiopurine S-Methyltransferase. *Clin.Pharmacol.Ther.*, **112**, 808-816.
13. Lin,S., Shi,Q., Nix,F.B., Styblo,M., Beck,M.A., Herbin-Davis,K.M., Hall,L.L., Simeonsson,J.B. and Thomas,D.J. (2002) A novel S-adenosyl-L-methionine:arsenic(III) methyltransferase from rat liver cytosol. *J Biol.Chem.*, **277**, 10795-10803.
14. Drozak,J., Piecuch,M., Poleszak,O., Kozlowski,P., Chrobok,L., Baelde,H.J. and de Heer E. (2015) UPF0586 Protein C9orf41 Homolog Is Anserine-producing Methyltransferase. *J Biol.Chem.*, **290**, 17190-17205.
15. Russell,D.A., Chau,M.K., Shi,Y., Levasseur,I.N., Maldonato,B.J. and Totah,R.A. (2023) METTL7A (TMT1A) and METTL7B (TMT1B) are responsible for alkyl S-thiol methyl transferase activity in liver. *Drug Metab Dispos.*, **51**, 1024-1034.
16. Maldonato,B.J., Russell,D.A. and Totah,R.A. (2021) Human METTL7B is an alkyl thiol methyltransferase that metabolizes hydrogen sulfide and captopril. *Sci.Rep.*, **11**, 4857.

Supplementary Figure 1. Zoomed-in version of Fig. 5B.

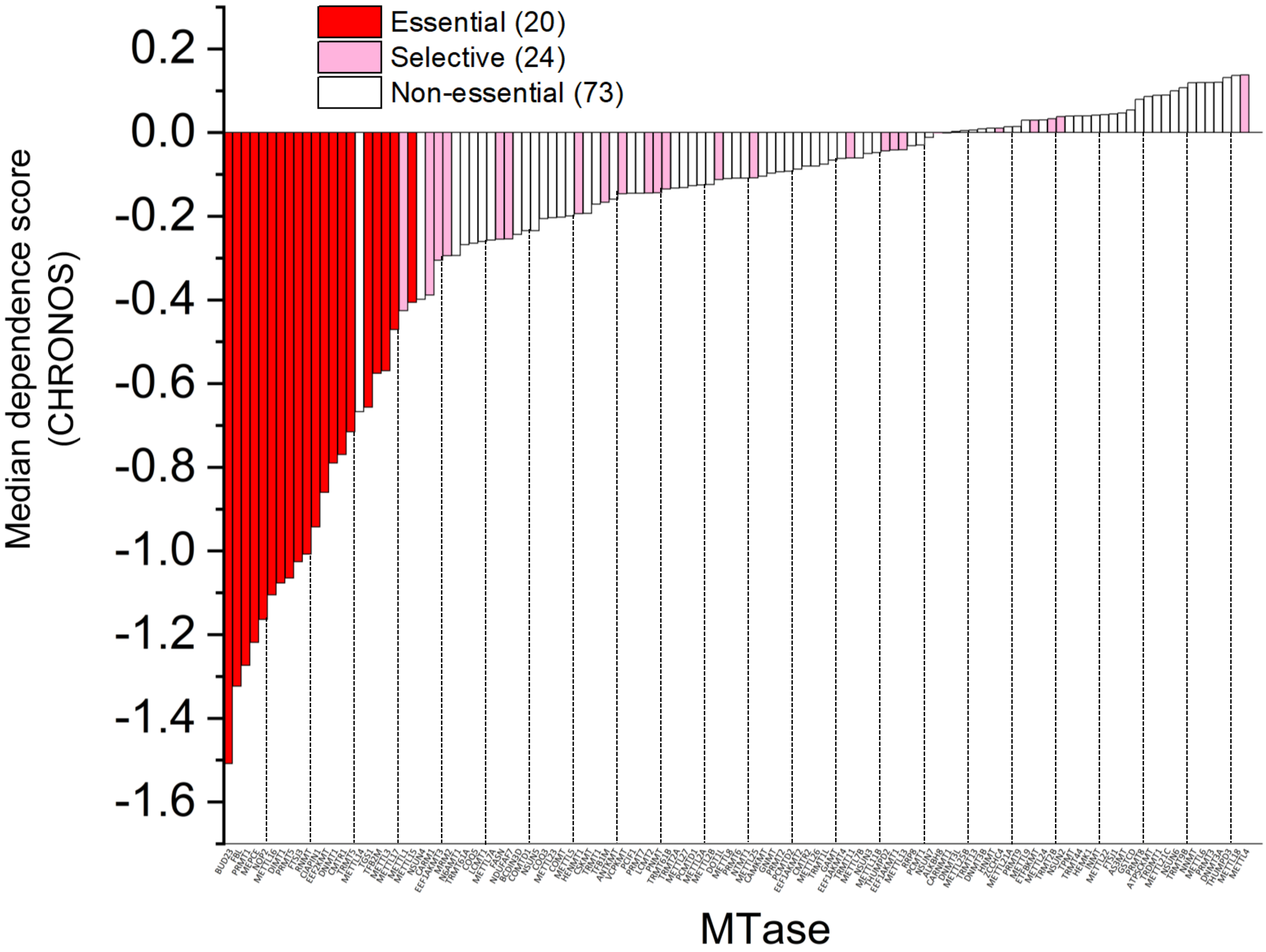

Supplement: gkae816_Supplemental_File [file gkae816_supplemental_file.pdf]
